# Reactions of an Osmium–Hexahydride Complex with 2-Butyne and 3-Hexyne and Their Performance in the Migratory Hydroboration of Aliphatic Internal Alkynes

**DOI:** 10.1021/acs.organomet.2c00338

**Published:** 2022-08-31

**Authors:** Juan C. Babón, Miguel A. Esteruelas, Ana M. López, Enrique Oñate

**Affiliations:** Departamento de Química Inorgánica, Instituto de Síntesis Química y Catálisis Homogénea (ISQCH), Centro de Innovación en Química Avanzada (ORFEO-CINQA), Universidad de Zaragoza-CSIC, 50009 Zaragoza, Spain

## Abstract

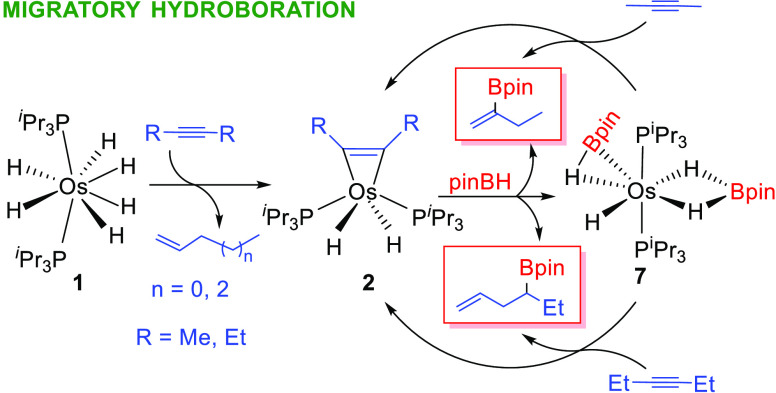

Reactions of the hexahydride OsH_6_(P^i^Pr_3_)_2_ (**1**) with 2-butyne and 3-hexyne and the behavior of the resulting species toward pinacolborane (pinBH) have been investigated in the search for new hydroboration processes. Complex **1** reacts with 2-butyne to give 1-butene and the osmacyclopropene OsH_2_(η^2^-C_2_Me_2_)(P^i^Pr_3_)_2_ (**2**). In toluene, at 80 °C, the coordinated hydrocarbon isomerizes into a η^4^-butenediyl form to afford OsH_2_(η^4^-CH_2_CHCHCH_2_)(P^i^Pr_3_)_2_ (**3**). Isotopic labeling experiments indicate that the isomerization involves Me-to-C_Os_ hydrogen 1,2-shifts, which take place through the metal. The reaction of **1** with 3-hexyne gives 1-hexene and OsH_2_(η^2^-C_2_Et_2_)(P^i^Pr_3_)_2_ (**4**). Similarly to **2**, complex **4** evolves to η^4^-butenediyl derivatives OsH_2_(η^4^-CH_2_CHCHCHEt)(P^i^Pr_3_)_2_ (**5**) and OsH_2_(η^4^-MeCHCHCHCHMe)(P^i^Pr_3_)_2_ (**6**). In the presence of pinBH, complex **2** generates 2-pinacolboryl-1-butene and OsH{κ^2^-*H*,*H*-(H_2_Bpin)}(η^2^-HBpin)(P^i^Pr_3_)_2_ (**7**). According to the formation of the borylated olefin, complex **2** is a catalyst precursor for the migratory hydroboration of 2-butyne and 3-hexyne to 2-pinacolboryl-1-butene and 4-pinacolboryl-1-hexene. During the hydroboration, complex **7** is the main osmium species. The hexahydride **1** also acts as a catalyst precursor, but it requires an induction period that causes the loss of 2 equiv of alkyne per equiv of osmium.

## Introduction

Understanding the behavior of transition-metal polyhydride complexes is one of the challenges in chemistry. The presence of metal–hydrogen bonds of different nature offers interesting chemical opportunities,^1^ as is evident in their ability to activate one of the widest ranges of σ-bonds.^2^ This property allows them to be relevant in a variety of fields including materials science,^3^ energy and environment,^4^ or organic synthesis based on metal-mediated catalysis.^5^ Thus, for example, this type of compounds are catalysts or catalyst precursors for some 40 different classes of organic reactions; the vast majority of them involve some σ-bond activation elemental step, in accordance with their tendency to activate σ-bonds.

The main fact explaining such versatility is probably the variety of roles that the coordinate hydrogen atoms can play during the catalysis. They can be transferred as a proton^6^ or hydride,^7^ undergo reductive elimination with other co-ligand to generate coordination vacancies,^8^ insert unsaturated organic molecules to afford organic ligands with a rich reactivity,^9^ promote C–H bond heterolytic activation acting as an internal Brønsted base,^2^ or even cooperate with the metal in the coordination of acidic ligands such as boranes.^10^ The latter are the main reagents in hydroboration and borylation reactions, processes of great current relevance because of the synthetic importance of organoboranes in organic chemistry.^11^

Several polyhydride complexes of rhenium and metals of the iron triad promote the hydroboration of olefins,^12^ alkynes,^13^ nitriles,^14^ N-heterocycles,^15^ and CO_2_.^16^ In addition, trihydride–iridium(III) complex IrH_3_{κ^3^-*P*,*O*,*P*-[xant(P^i^Pr_2_)_2_]} (xant(P^i^Pr_2_)_2_ = 9,9-dimethyl-4,5-bis(diisopropylphosphino)xanthene) catalyzes the borylation of arenes.^17^ Alkyne hydroboration is of special interest as is the most straightforward procedure to prepare useful alkenylborane synthetic intermediates. Polyhydrides for this catalysis have focused on the use of dihydride-(Kubas type-dihydrogen)-iron(II) and -ruthenium(II) complexes for the Z-hydroboration of terminal alkynes with pinacolborane (pinBH), while avoiding internal alkynes although the hydroboration of these substrates is attracting great attention with other families of catalysts.^18^

**Scheme 1:**
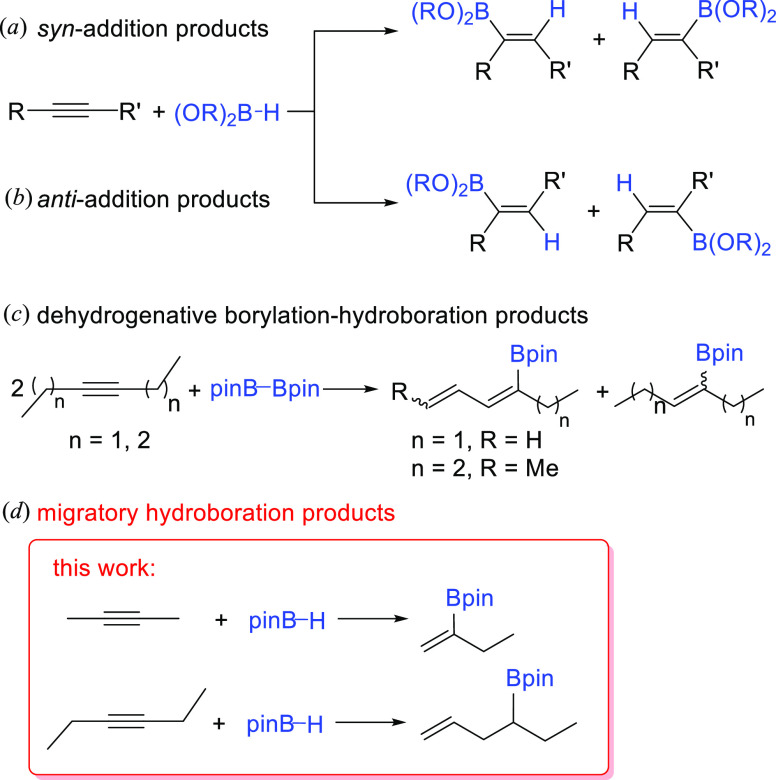
Different Products Resulting from Hydroboration and Dehydrogenative Borylation of Internal Alkynes

**Scheme 2:**
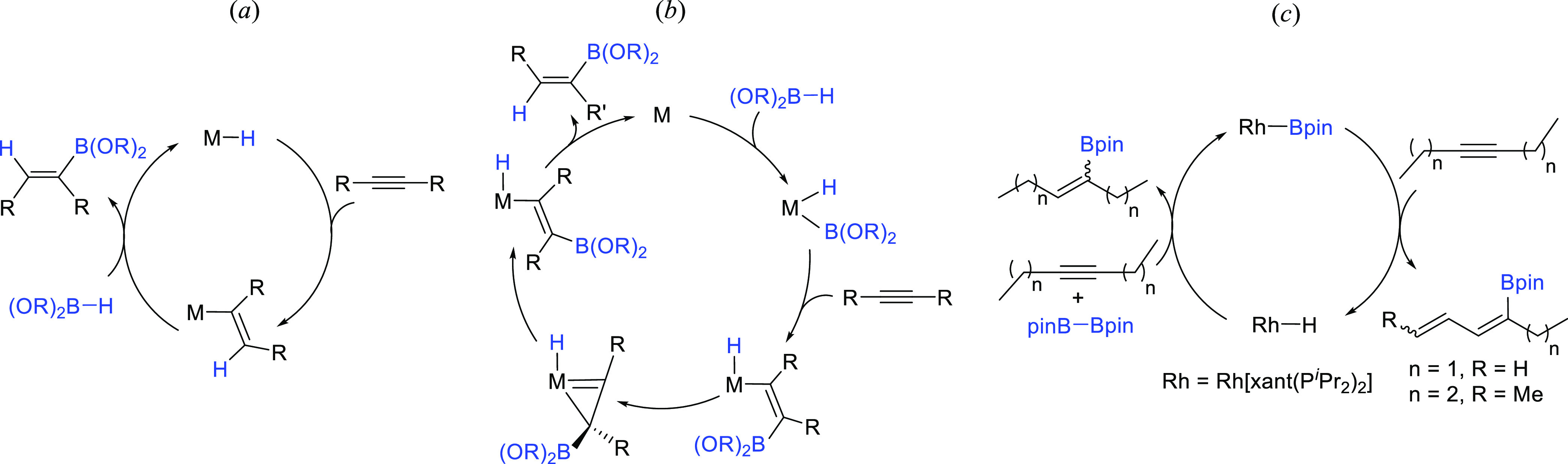
Mechanisms for the Hydroboration and Dehydrogenative Borylation of Internal Alkynes

Metal-catalyzed hydroboration of internal alkynes can produce four different alkenylborane products, resulting from syn- and anti-BH-addition (a^19^ and b^20^ in Scheme 1). The formation of one or the other depends upon the nature of the catalyst (Scheme 2). The syn-products are the most frequent and are particularly favored when the catalyst carries a hydride ligand, which provides metal-(E-alkenyl) intermediates. The reaction of the latter with the borane completes the catalysis (a in Scheme 2). In contrast, the anti-products are common for catalysts with boryl ligands. Insertion of the alkyne in the metal–boron bond affords metal-(E-borylalkenyl) intermediates. A boryl group at the C_β_ atom of the C–C double bond favors the E-to-Z isomerization of the alkenyl moiety,^21^ which is the key to the appearance of the anti-products.^22^ The boryl group lowers the activation energy to the formation of the metalacyclopropene responsible for the isomerization.^21^ Once Z-stereochemistry is achieved at the C–C double bond, reductive elimination involving the hydride and borylalkenyl ligands leads to the anti-products (b in Scheme 2). In addition, it has been recently observed that 3-hexyne and 4-octyne undergo rhodium-mediated dehydrogenative borylation–hydroboration with B_2_pin_2_, to give equimolecular mixtures of conjugated boryldienes and borylolefins (c in Scheme 1).^23^ The mixtures, which also contain the respective E and Z isomers, result from the addition of the B–B bond of the diborane to different molecules of alkyne and the hydride transfer from one to the other. Complexes Rh(Bpin){κ^3^-*P*,*O*,*P*-[xant(P^i^Pr_2_)_2_]} and RhH{κ^3^-*P*,*O*,*P*-[xant(P^i^Pr_2_)_2_]} collaborate to perform both borylations in a sequential and cyclic manner (c in Scheme 2). The rhodium(I)-boryl derivative promotes the stoichiometric dehydrogenative borylation to afford mixtures of E and Z boryldiene isomers and the rhodium(I)–hydride complex. The latter is responsible for the stoichiometric hydroboration, which yields the E and Z borylolefins and regenerates the rhodium–boryl compound to initiate the cycle again.

Hexahydride complex OsH_6_(P^i^Pr_3_)_2_ is a prominent member within the family of polyhydride derivatives,^24^ with a rich stoichiometric and catalytic reactivity and use as starting material to prepare compounds of interest in material science. It activates a vast range of σ-bonds,^2,25^ coordinates boranes stabilizing different bonding modes,^26^ promotes uncommon reactions as the metathesis between E–C(sp*^n^*) and H–C(sp^3^) σ-bonds (E = Si, Ge; *n* = 2, 3)^27^ is a precursor of osmium(II) and osmium(IV) phosphorescent emitters,^28^ and catalyzes a variety of organic transformations such as hydrogenation of nitriles to symmetrical and asymmetrical secondary amines,^29^ hydrogen transfer from 2-propanol to unsaturated organic substrates,^24^ dihydroboration of nitriles,^14^ deuteration of pyridines,^30^ hydration of nitriles,^31^ Tishchenko dimerization of cyclohexanecarboxaldehyde and benzaldehyde, and aldol-Tishchenko trimerization of isobutyraldehyde.^32^ Our interest in taking a further step in understanding the behavior of this fascinating polyhydride prompted us to study its reactivity toward internal alkynes such as 2-butyne and 3-hexyne, in the search of new hydroboration reactions. This paper describes the research steps taken to discover the migratory hydroboration of both alkynes (d in Scheme 1).

## Results and Discussion

### Metalacyclopropene and Butenediyl Compounds

**Scheme 3:**
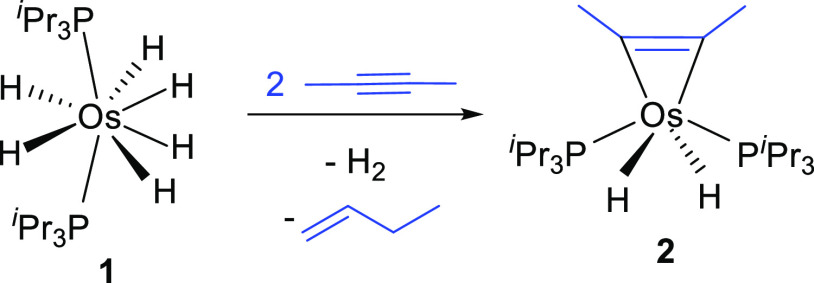
Reaction of 1 with 2-Butyne

Treatment of polyhydride complex OsH_6_(P^i^Pr_3_)_2_ (**1**) solutions, in toluene, with 2 equiv of 2-butyne, at 50 °C, for 18 h produces the release of 1 equiv of H_2_, the migratory hydrogenation^33^ of 1 equiv of alkyne to 1-butene, and the quantitative formation (according to the ^31^P{^1^H} NMR spectrum of the reaction crude) of the dihydride derivative OsH_2_(η^2^-C_2_Me_2_)(P^i^Pr_3_)_2_ (**2**), which was isolated as an orange solid in 78% yield (Scheme 3) and characterized by X-ray diffraction analysis.

**Figure 1:**
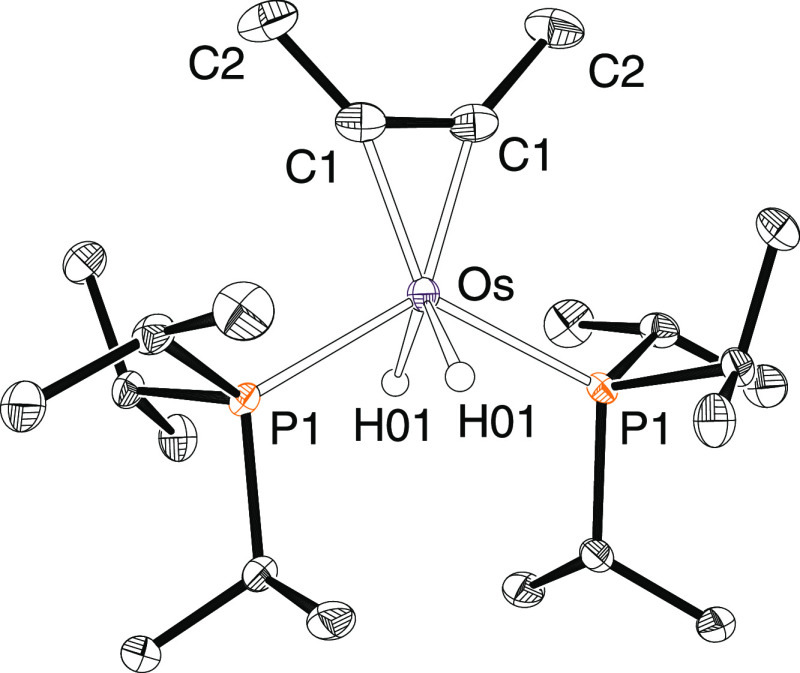
Molecular structure of **2** with ellipsoids at the 50% probability level. Hydrogen atoms are omitted for clarity (except for hydride ligands). Selected bond distances (Å) and angles (°) for the X-ray structure and DFT-optimized (in square brackets): Os–C(1) = 1.985(3) [2.01], Os–P(1) = 2.3155(8) [2.37], C(1)–C(1) = 1.308(8) [1.34], C(1)–C(2) = 1.501(5) [1.49]; P(1)–Os–P(1) = 123.99(4) [119.8], H(01)–Os–H(01) = 106(3) [112.4], C(1)–C(1)–C(2) = 134.1(2) [136.3].

The structure (Figure 1) resembles that of the known complex OsH_2_Cl_2_(P^i^Pr_3_)_2_^24^ with the CMe units of the hydrocarbon occupying the positions of the chloride ligands. Six-coordinate structures that deviate significantly from the typical octahedron are characteristic for complexes of d^4^-ions bearing a π-donor ligand, in particular for osmium(IV) hydride derivatives.^34^ The distortion destabilizes a half-occupied orbital of the *t*_2*g*_ set but stabilizes the other one, resulting in a diamagnetic species. The process produces a partial cancelation of the electron deficiency at the metal center, which receives additional electron density from the π-donor ligand via the corresponding π-bond. Such disposition in the case of **2** indicates that the hydrocarbon, which acts as a 4e donor ligand,^35^ undergoes the oxidative addition of one of its π-bonds to the metal center, whereas the other works to cancel the metal electron deficiency. In agreement with the oxidative addition of one of the π-bonds of the alkyne, the C(1)–C(1) distance of 1.308(7) Å and the C(1)–C(1)–C(2) angle of 134.4(2)° (1.34 Å and 136.3° in the density functional theory (DFT)-optimized structure) are consistent with the formation of a metalacyclopropene. The coordinated carbon atoms give rise to a triplet (^2^*J*_C–P_ = 5.9 Hz) at 168.7 ppm in the ^13^C{^1^H} NMR spectrum, in toluene-*d*_8_, at room temperature. The most notable resonance in the ^1^H NMR spectrum is a triplet at −19.79 ppm, with an H–P coupling constant of 33.1 Hz, corresponding to the hydride ligands. The ^31^P{^1^H} NMR spectrum contains a singlet at 48.9 ppm.

**Scheme 4:**
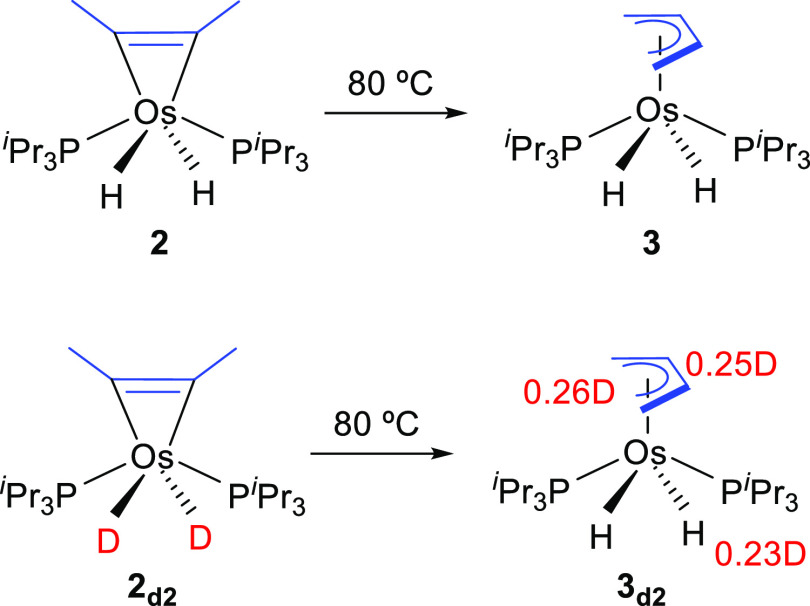
Isomerization of **2** into **3**

Complex **2** is quantitatively transformed into the butenediyl isomer OsH_2_(η^4^-CH_2_CHCHCH_2_)(P^i^Pr_3_)_2_ (**3**) after 24 h, at 80 °C, in toluene. The isomerization involves osmium-mediated 1,2-hydrogen shifts from the methyl substituents to the coordinated atoms of the metalacyclopropene unit. In agreement with the participation of the metal in the process, a complete deuterium distribution between the metal center and the butenediyl positions was observed in the deuterated **3**_**d**_**2**__ species, when the dideuteride derivative OsD_2_(η^2^-C_2_Me_2_)(P^i^Pr_3_)_2_ (**2**_**d**_**2**__) was used as starting point (Scheme 4).

**Scheme 5:**
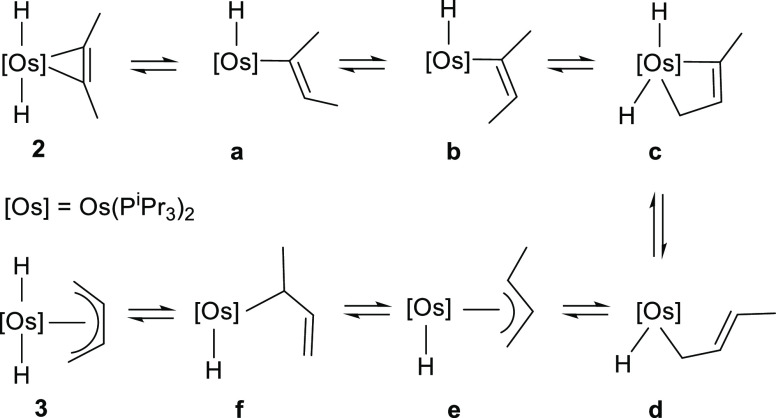
Mechanism for the Isomerization of **2** into **3**

The isomerization can be rationalized according to Scheme 5. The initial migration of one of the hydride ligands to a coordinated carbon atom should give the E-alkenyl intermediate **a**, which could undergo E-to-Z isomerization of the alkenyl group to afford **b**. The subsequent C–H bond activation of the methyl substituent at the C_β_-atom of the C–C double bond disposed syn to the metal center should lead to the metalacycle **c**, which would evolve into **d** by reductive migration of one of the hydride ligands to the C(sp^2^)-metalated carbon atom. Thus, the equilibrium between allyl species could convert **d** into **f** via **e**. A β-hydrogen elimination on the methyl substituent at the σ-allyl ligand should finally yield **3**.

**Figure 2:**
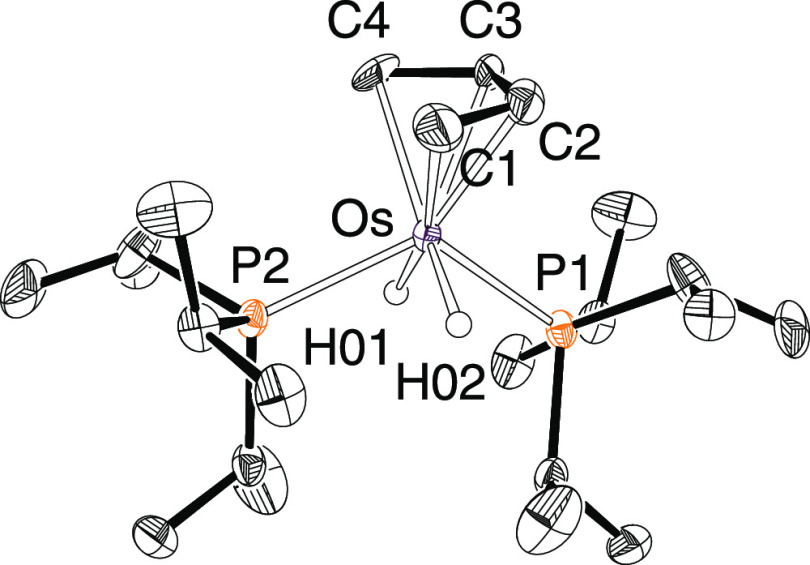
Molecular structure of **3** with ellipsoids at the 50% probability level. Hydrogen atoms are omitted for clarity (except for hydride ligands). Selected bond distances (Å) and angles (°) for the X-ray structure and DFT-optimized (in square brackets): Os–C(1) = 2.235(11) [2.27], Os–C(2) = 2.197(12) [2.21], Os–C(3) = 2.192(12) [2.21], Os–C(4) = 2.235(11) [2.27], Os–P(1) = 2.2535(15) [2.33], Os–P(2) = 2.3984(15) [2.37], Os–H(01) = 1.590(10) [1.64], Os–H(02) = 1.592(10) [1.64], C(1)–C(2) = 1.43(2) [1.43], C(2)–C(3) = 1.43(2) [1.43], C(3)–C(4) = 1.46(2) [1.44]; P(1)–Os–P(2) = 119.22(7) [119.2], H(01)–Os–H(02) = 110(6) [120.0], C(1)–C(2)–C(3) = 116.2(14) [116.9], C(2)–C(3)–C(4) = 118(2) [117.4].

Complex **3** was isolated as a white solid in 83% yield and characterized by X-ray diffraction analysis. The structure (Figure 2) proves the isomerization of the hydrocarbon. The arrangement of ligands around the metal center can be described as a four-legged piano stool, with the butenediyl at the seat, whereas the hydride and phosphine ligands occupy the legs in an alternated manner. This disposition is usual in cations of the class [Os(η^5^-C_5_R_5_)H_2_(PR_3_)_2_]^+^ ^36^ and suggests a +4 oxidation state for the metal center. In accordance with these species, the P(1)–Os–P(2) and H(01)–Os–H(02) angles are 119.22(7) and 110(6)° (119.2 and 120° in the DFT-optimized structure), respectively. The butenediyl coordinates with Os–C distances in the range 2.192(12)–2.235(11) Å (2.21–2.27 Å in the optimized structure). The butenediyl C–C distances of 1.43(2)–1.46(2) Å (1.43–1.44 in the DFT-optimized structure) are essentially the same and point out a very light partial double-bond character of the bonds between the carbons. In this context, it should be noted that the delocalized electron density between the four carbon atoms is that corresponding to one bond (2e). In toluene-*d*_8_, at room temperature, the butenediyl rotates around an osmium-butenediyl axis as revealed by the ^31^P{^1^H} NMR spectrum, which displays a singlet at 37.9 ppm for the inequivalent phosphines. In the ^1^H NMR spectrum, the resonance corresponding to the hydride ligands appears at −13.65 ppm as a triplet with an H–P coupling constant of 32.0 Hz, which is consistent with the relative cisoid disposition of the hydride and phosphine ligands in the four legs of the stool. Characteristic resonances of the butenediyl in this spectrum are three signals at 4.53 (CH), 2.07, and −0.52 (CH_2_) ppm, which fit with resonances at 68.4 (CH) and 25.5 (CH_2_) in the ^13^C{^1^H} NMR spectrum.

**Scheme 6:**
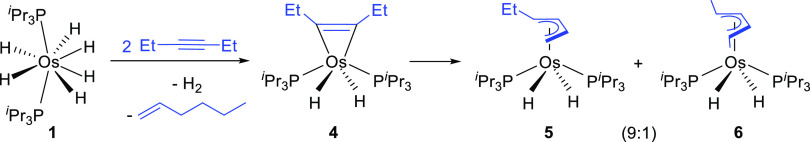
Reaction of 1 with 3-Hexyne

3-Hexyne displays similar behavior to 2-butyne; it undergoes migratory hydrogenation to 1-hexene and coordinates to the resulting metal center to afford the counterpart metalacyclopropene complex OsH_2_(η^2^-C_2_Et_2_)(P^i^Pr_3_)_2_ (**4**), although the hydrocarbon of the latter appears to have a smaller activation energy for the isomerization into butenediyl than in **2**. The formation rate of **4** and the rate of isomerization are comparable. As a consequence, the isomerization starts before the complete formation of **4**. Furthermore, the isomerization affords two products, the ethylbutenediyl derivative OsH_2_(η^4^-CH_2_CHCHCHEt)(P^i^Pr_3_)_2_ (**5**) and the dimethylbutenediyl species OsH_2_(η^4^-MeCHCHCHCHMe)(P^i^Pr_3_)_2_ (**6**), the first of them being the main one in a 9:1 molar ratio (Scheme 6). The formation of both butenediyls takes place by metal-mediated ethyl-to-C_Os_ 1,3-hydrogen shifts. Complex **5** results from migrations from the CH_2_ and CH_3_ units of an ethyl substituent, while the generation of the minor isomer **6** involves the CH_2_ groups of both ethyl substituents.

Complexes **4–6** were fully characterized by NMR spectroscopy. The spectra of **4** (Figures S14–S17) agree well with those of **2**; the hydride resonance in the ^1^H spectrum appears at −18.14 (^2^*J*_H–P_ = 33.1 Hz) ppm, whereas the ^13^C{^1^H} spectrum shows the signal due to the coordinated C atoms at 176.4 (^2^*J*_C–P_ = 6.3 Hz) ppm. The ^31^P{^1^H} spectrum contains a singlet at 48.2 ppm. Spectra of the butenediyl derivative **5** (Figures S18–S25) are consistent with the asymmetry introduced in the molecule by the ethyl substituent of the butenediyl, which converts both hydrides and phosphines into inequivalent ligands. Furthermore, the substituent makes the rotation of the butenediyl around the osmium-butenediyl axis difficult. Thus, ^1^H and ^31^P{^1^H} spectra are temperature-dependent. At 233 K, the hydride ligands generate two doublets of doublets (^2^*J*_H–P_ = 36.6 and 27.3 Hz) at −13.25 and −14.40 ppm, in the ^1^H, whereas an AB spin system (*J*_AB_ = 86 Hz, Δ*ν* = 979 Hz) centered at 28.7 ppm is observed in the ^31^P{^1^H}. In contrast to **5**, only the phosphine ligands are inequivalent in **6**, whereas the presence of two substituents in the butenediyl prevents its rotation, even at room temperature. Thus, the ^1^H shows a doublet of doublets (^2^*J*_H–P_ = 36.8 and 28.2 Hz) at −14.11 ppm, due to the equivalent hydrides (Figure S20), whereas the inequivalent phosphines give rise to an AB spin system (*J*_AB_ = 89 Hz, Δ*ν* = 1145 Hz) at 27.0 ppm in the ^31^P{^1^H} (Figure S22).

### Reaction of the Metalacyclopropene Compound 2 with Pinacolborane

**Scheme 7:**
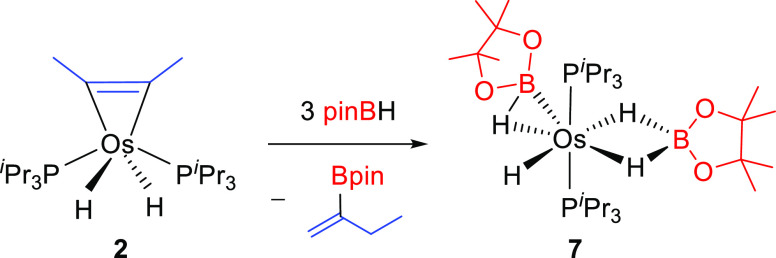
Reaction of **2** with Pinacolborane

Having studied the access of the alkynes to the metal center and analyzed the alkyne–osmium interaction, we decided to investigate the entry of the other component of the hydroboration process, pinBH. Thus, we carried out the reaction of the metalacyclopropene complex **2** with the borane. Treatment of solutions of this complex, in toluene, with 5 equiv of pinBH, at room temperature for 3 h leads to the dihydrideborate-osmium(II)-(elongated σ-borane) derivative OsH{κ^2^-*H*,*H*-(H_2_Bpin)}(η^2^-HBpin)(P^i^Pr_3_)_2_ (**7**) and 2-pinacolboryl-1-butene, as a result of the migratory hydroboration of the coordinated hydrocarbon and the coordination of two borane molecules to the resulting dihydride fragment (Scheme 7). Complex **7** was isolated as a white solid in 71% and characterized by X-ray diffraction analysis.

**Figure 3:**
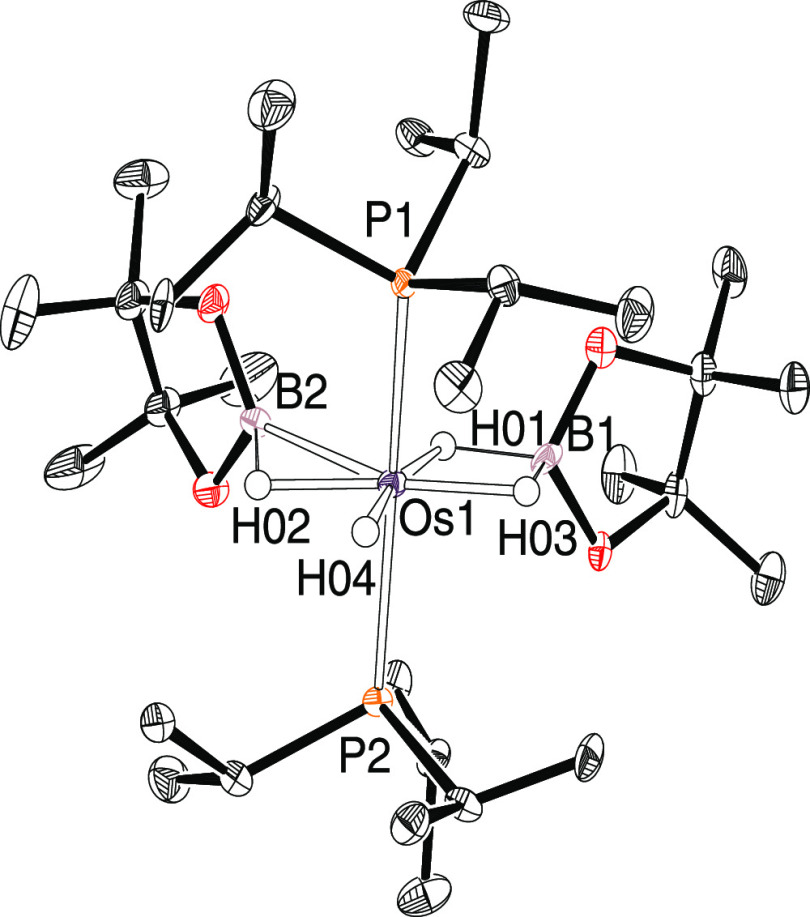
Molecular structure of **7** with ellipsoids at the 50% probability level. Hydrogen atoms are omitted for clarity (except for hydrides). Selected bond distances (Å) and angles (°) for the X-ray and DFT-optimized (in square brackets) structures: Os–B(1) = 2.222(10) [2.24], Os–B(2) = 2.132(9) [2.14], Os–H(01) = 1.587(10) [1.76], Os–H(02) = 1.585(10) [1.69], Os–H(03) = 1.585(10) [1.74], Os–H(04) = 1.587(10) [1.66], Os–P(1) = 2.3522(18) [2.36], Os–P(2) = 2.3624(18) [2.36], B(1)–H(01) = 1.28(9) [1.41], B(1)–H(03) = 1.39(10) [1.44], B(2)–H(02) = 1.44(10) [1.48]; P(1)–Os–P(2) = 160.69(6) [162.1], H(01)–Os–H(04) = 167(5) [172.6], H(01)–Os–H(03) = 73(5) [78.9], H(02)–Os–H(04) = 75(5) [72.6], H(03)–Os–H(04) = 95(5) [93.9], H(01)–Os–B(2) = 75(3) [72.3], H(02)–Os–B(2) = 42(4) [43.7].

The structure (Figure 3) proves the presence of two borane molecules at the metal center, coordinated in a different manner: dihydrideborate (B(1)) and elongated σ-borane (B(2)). The dihydrideborate ligand acts as κ^2^-*H*,*H*-chelate with a bite angle of 73(5)°; the separation between its boron and the osmium atom of 2.222(10) (2.24 Å in the DFT-optimized structure) compares well with those found in other crystallographically characterized related osmium complexes.^37^ The coordination of the B(2)–H(02) bond of the second borane molecule gives rise to a metal–(σ-bond) interaction weaker than those found in other elongated σ-borane complexes as proved by the B(2)–H(02) distance of 1.44(10) Å (1.48 Å in the DFT-optimized structure), which is between 0.1 and 0.2 Å shorter than the B–H distances reported for complexes OsHCl(η^2^-HBR_2_){κ^3^-*P*,*O*,*P*-[xant(P^i^Pr_2_)_2_]} (1.60–1.69 Å)^38^ and Rh(η^5^-C_5_Me_5_)(Bpin)_2_(η^2^-HBpin) (1.53(2) and 1.69(3) Å).^39^ The B(2)–H(02) bond length compares well with the B–H distance found in the iridium complex Ir{κ^3^-*P*,*C*,*P*-[C_6_H_3_-1,3-OP^t^Bu_2_]}(η^2^-HBpin) (1.47(6) Å).^40^ The B–H bonds of the dihydrideborate and elongated σ-borane groups are located, together with a hydride ligand, in a perpendicular plane to an ideal direction defined by the mutually trans-disposed phosphine ligands and the osmium atom (P(1)–Os–P(2) = 160.69(6)°), in the expected octahedron for a six-coordinate d^6^-species.

**Figure 4:**
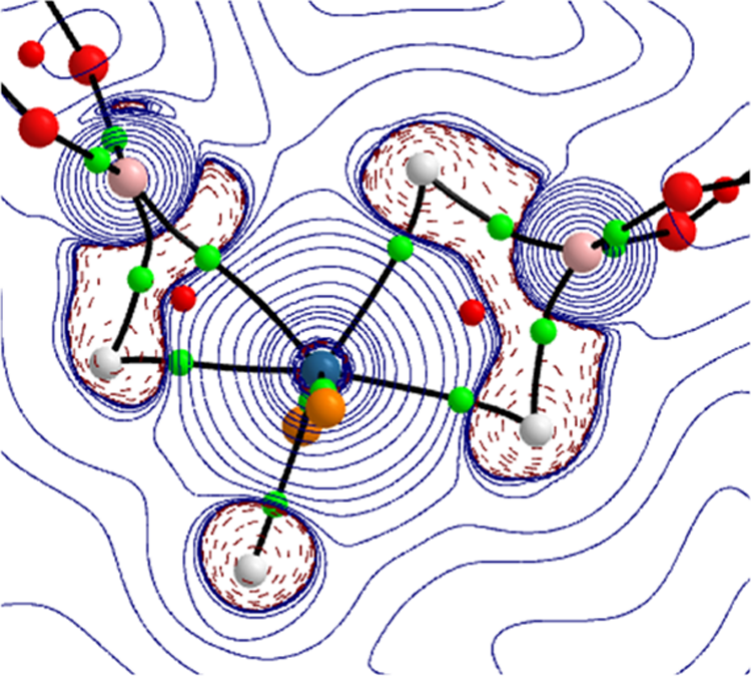
Contour line diagram ∇^2^ρ(*r*) for complex **7** in the OsH_4_B_2_ plane. The black lines connecting the atomic nuclei are the bond paths, while the small green and red spheres designate the corresponding bond and ring critical points, respectively.

Atoms in Molecules (AIM) analysis of **7** confirmed its dihydrideborate-osmium(II)-(elongated σ-borane) nature (Figure 4). In accordance with other dihydrideborate complexes,^37c^ the four-membered cycle formed by the osmium atom and the H_2_B moiety displays two Os–H and two B–H bond critical points associated with one OsHHB ring critical point. The metal–(σ-bond) interaction shows the characteristic triangular topology of elongated σ-bonds acting as 2e donor ligands,^38^ which is defined by bond critical points located between the involving atoms, associated with bond paths running between them, and all complemented by a ring critical point. The coordination mode of the dihydrideborate group in **7** contrasts with that found in the dihydridecatecholborate counterpart derivative OsH(η^3^-H_2_Bcat)(η^2^-HBcat)(P^i^Pr_3_)_2_, which has been described as bis(elongated σ) on the basis an AIM analysis and the energy decomposition analysis (EDA) method.^26^

The coordinated hydrogen atoms of the dihydrideborate and borane groups and the hydride ligand exchange their positions in solution. Thus, the four inequivalent nuclei give rise to only one resonance, at −10.65 ppm, in the ^1^H NMR spectrum, in toluene-*d*_8_, at room temperature. The exchange is thermally activated. As a consequence, the spectrum is temperature-dependent. About 233 K, the resonance decoalesces, and between 213 and 183 K, three signals at −9.44, −9.64, and −11.88 ppm are clearly observed. The exchange is also supported by the ^11^B spectrum at room temperature, which shows a broad resonance at 37.9 ppm for both borane and dihydrideborate. In contrast to the ^1^H spectrum, the ^31^P{^1^H} is temperature-invariant, displaying a singlet at 33.7 ppm in agreement with the equivalence of the phosphines.

**Scheme 8:**
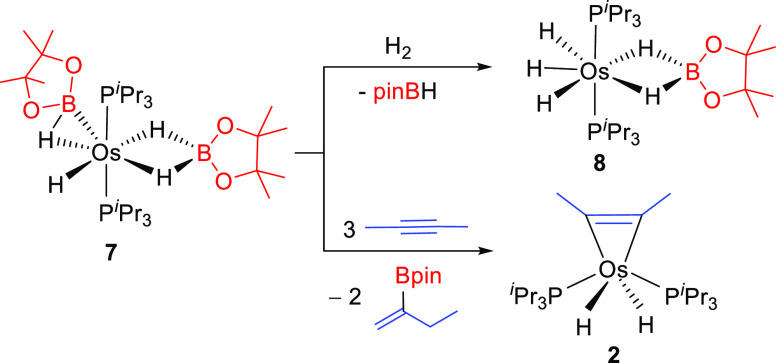
Reactions of **7** with Hydrogen or 2-Butyne

The interaction between the σ-bond of the borane and the metal center is certainly weak. Thus, in spite of the saturated character of **7**, it reacts with a weak Lewis base such as molecular hydrogen. The H_2_ molecule displaces the elongated σ-borane ligand from the metal coordination sphere to give the previously reported trihydride-osmium(IV)-dihydrideborate derivative OsH_3_{κ^2^-*H*,*H*-(H_2_Bpin)}(P^i^Pr_3_)_2_ (**8**).^26^ This finding explains why complex **7** is inaccessible by direct reaction between the hexahydride **1** and pinBH and why complex **8** is the isolated species. The lability of the elongated σ-borane ligand was certainly a promising feature of **7**, given the presence of an additional molecule of borane coordinated in the dihydrideborate form. Such lability stimulated our interest to know the potential performance of **7** in the alkyne hydroboration and prompted us to study the reaction between this bis-borylated complex and alkyne in excess. The addition of 5 equiv of 2-butyne to **7**, in tolune-*d*_8_, at room temperature gives 2 equiv of 2-pinacolboryl-1-butene and regenerates **2** (Scheme 8).

### Catalytic Hydroboration of 2-Butyne and 3-Hexyne

The reaction of **2** with pinBH to give 2-pinacolboryl-1-butene and **7** (Scheme 7) and the reaction of the latter with 2-butyne to afford 2-pinacolboryl-1-butene again and regenerate **2** (Scheme 8) form a cycle. Accordingly, complex **2** catalyzes the migratory hydroboration of 2-butyne to 2-pinacolboryl-1-butene. The reaction was performed in toluene, at 60 °C, using a 5 mol % of catalyst and an alkyne/borane molar ratio of 1:1.5. The borylated olefin was selectively formed after 2 h (91%) and isolated in 82% yield (d in Scheme 1). Under the same conditions, 3-hexyne was transformed into a mixture of the migratory hydroboration product 4-pinacolboryl-1-hexene (85%, d in Scheme 1) and the syn-hydroboration product 3-pinacolboryl-3-hexene (14%, a in Scheme 1) after 3 h. The former was separated from the mixture by flash chromatography in silica gel, using hexane as eluent, and isolated pure in 77% yield.

Migratory hydrofunctionalization of internal alkynes catalyzed by transition-metal complexes is a scarcely explored class of reactions. It has been mainly focused on hydrosilylation and hydrogermylation processes promoted by cobalt-hydride compounds. Mechanistic details are even scarcer, with the reaction mechanisms being true black boxes.^41^

**Scheme 9:**
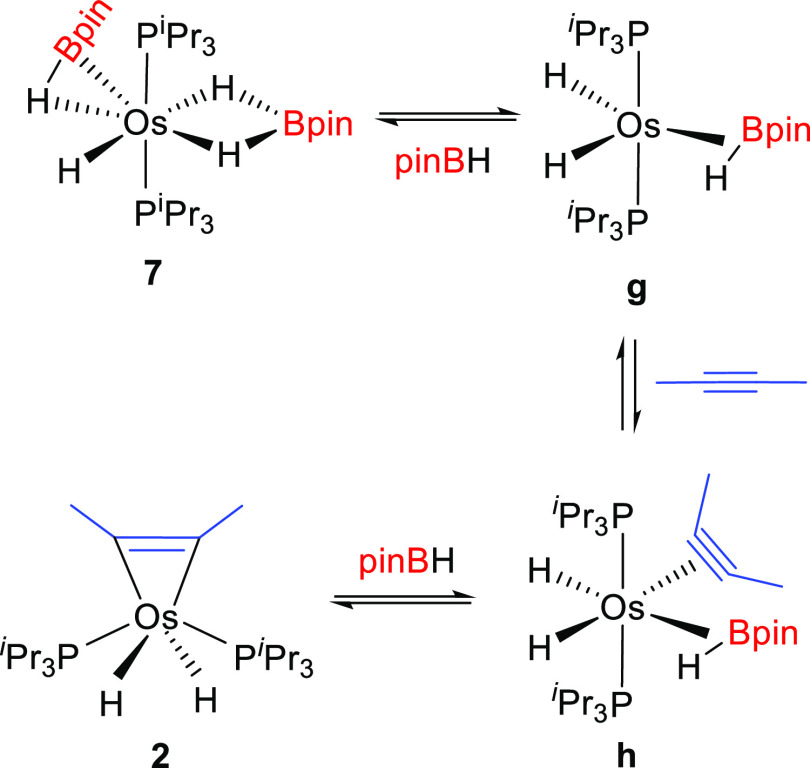
Formation of Catalytic Active Species

The ^1^H and ^31^P{^1^H} NMR spectra of the catalytic solutions revealed that complex **2** is rapidly and quantitatively transformed into **7**, which is the main metallic species while alkyne and borane are present in solution. Its incorporation into the catalytic cycle takes place by means of the dissociation of a borane molecule. In agreement with this, the hydroboration rate decreases by increasing the borane concentration; the catalysis is inhibited for borane:alkyne molar ratios >10. Previous DFT calculations about the dissociation of molecular hydrogen from **8** revealed that the interaction hydride-borane affording the dihydridoborate group is broken in the resulting unsaturated five-coordinate intermediate, which was described as the dihydride-osmium(II)-(elongated σ-borane) species OsH_2_(η^2^-HBpin)(P^i^Pr_3_)_2_ (**g**).^14^ Therefore, this is the species formed as a result of the dissociation of a borane molecule from **7** (Scheme 9). The coordination of the alkyne to intermediate **g** should lead to **h**. Related compounds with a carbonyl group instead of an alkyne ligand have been experimentally observed in equilibrium with dihydrideborate-osmium(II)-hydride derivatives.^37b^ Dihydride-osmium(II)-(elongated σ-borane) species like **h**, with a coordinated nitrile instead of a carbonyl group or an alkyne ligand, have been also proposed as key intermediates for the hydroboration of aliphatic nitriles, on the basis of DFT calculations.^14^ Intermediate **h** could be also formed by the addition of the borane to **2** and, since it contains both components of the hydroboration products, it should be the species responsible for the catalysis and thus the species connecting **2** and **7** (Scheme 9).

**Scheme 10:**
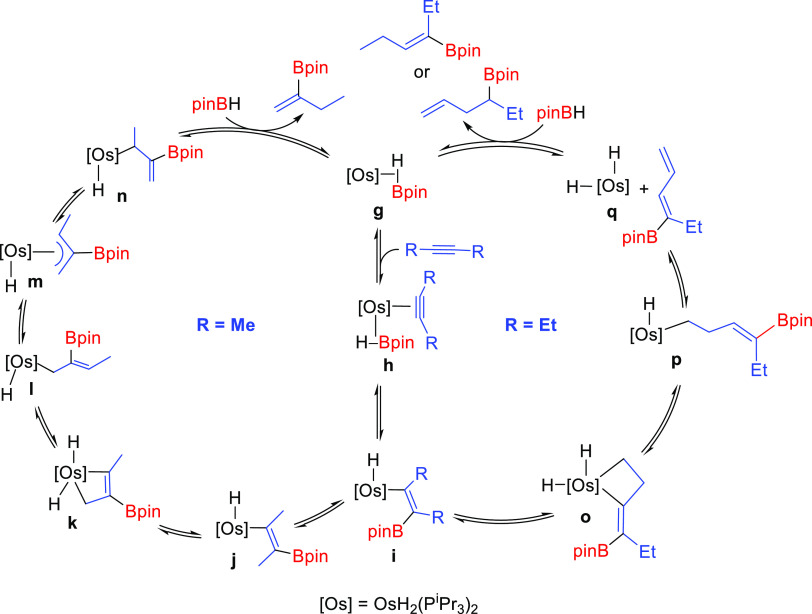
Proposed Catalytic Cycles

The catalysis can be rationalized according to Scheme 10. The insertion of the coordinated alkyne into the Os–B bond of **h** should give metal-(E-borylalkenyl) intermediates **i**, which would evolve in a different manner depending upon the size of the substituents at the C–C double bond. When the substituent is methyl (the 2-butyne case), the E-to-Z isomerization of the borylalkenyl group takes place. Such isomerization should lead to **j**. The unsaturated character of the metal center of the latter and its syn-disposition to the β-methyl group would favor the C–H bond activation of this substituent to give **k**. Intermediate **k** could generate the σ-allyl derivative **l** by reductive elimination of the alkenyl moiety. Once formed **l**, it should be transformed into the σ-allyl counterpart **n**, via the π-allyl **m**. Thus, in the presence of the borane, the reductive elimination of the σ-allyl could yield 2-pinacolboryl-1-butene and regenerate **g**. The bigger steric hindrance of ethyl with respect to methyl appears to prevent the E-to-Z isomerization of the borylalkenyl group of the corresponding intermediate **i**. Thus, for the 3-hexyne case, the C–H bond activation of the methyl group of the ethyl substituent at the C_α_ atom of the borylalkenyl group would lead to **o**, which could evolve into **p** by reductive elimination of the borylalkenyl moiety. A subsequent β-hydrogen elimination should give the unsaturated tetrahydride **q** and 4-pinocolboryl-1,3-hexadiene. The reduction of one of the C–C double bonds of the diene by the tetrahydride complex would afford the hydroboration products and a dihydride-metal fragment that could regenerate **g** by coordination of the borane. The reduction of the internal olefinic bond should yield the main product, 4-pinacolboryl-1-hexene, the terminal olefin resulting from the migratory hydroboration, while the reduction of the terminal C–C double bond should lead to 3-pinacolboryl-3-hexene. The latter could be also formed by reductive elimination from **i**.

In short, the formation of α-vinylborane or homoallylborane products depends on whether or not the borylalkenyl intermediate undergoes E-to-Z isomerization.

**Figure 5:**
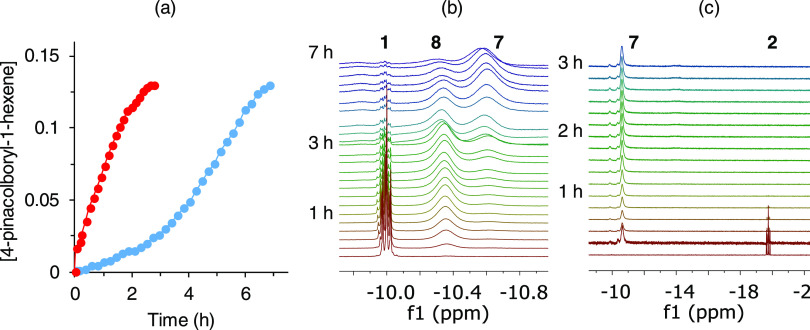
(a) Formation of 4-pinacolboryl-1-hexene by hydroboration of 3-hexyne (0.15 M) with pinBH (0.23 M) in the presence of **1** (blue •) and **2** (red •) (7.5 × 10^–3^ M), respectively, in toluene-*d*_8_ at 60 °C. (b) High-field region of the ^1^H NMR spectrum of the catalytic solutions using **1** as a catalyst precursor as a function of time. (c) High-field region of the ^1^H NMR spectrum of the catalytic solutions using **2** as catalyst precursor as a function of time.

Having achieved the migratory hydroboration of 2-butyne and 3-hexyne and analyzed the formation of the products, we decided to address the direct use of the hexahydride complex **1** as a catalyst precursor. Indeed, this polyhydride is also an efficient catalyst precursor for the migratory hydroboration of both alkynes, the main difference with regard to the reactions performed with the cyclopropane complex **2** is the additional formation of the terminal olefin, resulting from the migratory hydrogenation of the alkyne. The formed amount of this product is about twice the percentage of moles of the catalytic precursor used in the reaction (about 10% of alkyne). Figure 5a shows the formation of the product of the migratory hydroboration of 3-hexyne, 4-pinacolboryl-1-hexene, as a function of time in the presence of **1** and **2**, respectively. The formation of the borylated olefin in the presence of the hexahydride **1** displays an induction period, which is not observed when the precursor is the metalacyclopropene complex **2**. However, the slope of the line defining the reaction course is almost identical in both cases after the induction time. This finding suggests that both precursors give rise to the same active species. To confirm this conclusion and to understand the induction period, we analyzed the ^1^H NMR spectra of the catalytic solutions generated from **1** and **2**, in the high-field region, as a function of time (Figure 5b,c, respectively). As expected, the main osmium species is complex **7** in both cases. However, it is generated in a different way. Complex **2** is rapidly transformed into **7**, according to Scheme 9, via the spectroscopically undetected intermediates **g** and **h** (Figure 5c); it is not necessary for any induction period. In contrast, hexahydride complex **1** is first converted into **8**, which is transformed into **7** when the excess of hydrogen provided by **1** is consumed (Figure 5b). So, an induction period is necessary to eliminate two hydrogen molecules from the reaction medium.

In conclusion, hexahydride complex **1** is also an efficient catalyst precursor for the migratory hydroboration of internal alkynes but causes the loss of 2 equiv of alkyne per equiv of catalyst precursor, generating as a byproduct the olefin resulting from a migratory hydrogenation.

## Concluding Remarks

This study has revealed that the hexahydride complex OsH_6_(P^i^Pr_3_)_2_ induces the stoichiometric migratory hydrogenation of 2-butyne and 3-hexyne to afford the corresponding terminal olefins and a dihydride-osmium(II) fragment, which oxidatively adds one of the three bonds of the triple bond of a new alkyne molecule, to form formally unsaturated d^4^-dihydride-osmacyclopropene complexes. These compounds isomerize into (η^4^-butenediyl)-osmium(IV)-dihydride derivatives by hydrogen shifts from the substituents of the three-member ring to the carbon atoms of the ring. Isotopic labeling experiments indicate that the hydrogen shifts take place through the metal center and involve C–H bond activation processes.

Complexes dihydride-osmacyclopropene react with pinacolborane to produce α-vinylborane (2-butyne) or homoallylborane (3-hexyne) derivatives and the dihydrideborate-osmium(II)-(elongated σ-borane) compound OsH{κ^2^-*H*,*H*-(H_2_Bpin)}(η^2^-H-Bpin)(P^i^Pr_3_)_2_. In agreement with the formation of the borylated olefins, these dihydride–osmacylopropene complexes promote the migratory hydroboration of 2-butyne to 2-pinacolboryl-1-butene and 3-hexyne to 4-pinacolboryl-1-hexene. During the hydroboration process, the dihydrideborate-osmium(II)-(elongated σ-borane) compound is the main osmium species. The hexahydride complex is also a catalyst precursor for these reactions, but it needs an induction period to reach the maximum activity that causes the loss of 2 equiv of alkyne per equiv of osmium.

In summary transition-metal polyhydride complexes are promising catalyst precursors for migratory hydrofunctionalization reactions of internal aliphatic alkynes due to their ability to activate σ-bonds, in particular C–H.

## Experimental Section

### General Information

All reactions were carried out with exclusion of air at an argon/vacuum manifold using standard Schlenk tube or glovebox techniques. Complexes OsH_6_(P^i^Pr_3_)_2_ (**1**)^24^ and OsD_6_(P^i^Pr_3_)_2_ (**1**_d_6__)^25i^ were prepared according to the published methods. Instrumental methods used for characterization, X-ray information, and computational details are given in the Supporting Information. Chemical shifts (in ppm) are referenced to residual solvent peaks (^1^H, ^13^C{^1^H}), external H_3_PO_4_ (^31^P{^1^H}), or BF_3_·OEt_2_ (^11^B). Coupling constants are given in hertz.

#### Preparation of OsH_2_(η^2^-C_2_Me_2_)(P^i^Pr_3_)_2_ (**2**)

2-Butyne (152 μL, 1.93 mmol) was added over a solution of complex 1 (500 mg, 0.96 mmol) in toluene (2 mL). The mixture was heated at 50 °C for 18 h. After this time, the ^1^H NMR spectrum of reaction crude in toluene-d_8_ showed the formation of **2** along with 1-butene. Then, it was concentrated to dryness to give an orange oil. The addition of pentane (2 mL) at −78 °C afforded an orange solid that was washed with cold pentane (2 × 2 mL). Orange single crystals suitable for X-ray diffraction analysis were obtained from a saturated solution of **2** in pentane at −30 °C. Yield: 425 mg (78%). Anal. Calcd. for C_22_H_50_OsP_2_: C, 46.62; H, 8.89. Found: C, 46.27; H, 9.39. HR-MS (electrospray): *m*/*z* calcd for C_22_H_49_OsP_2_ [M – H]^+^ 567.2919; found 567.2903. IR (ATR, cm^–1^): ν(Os–H) 2136. ^1^H NMR (300.13 MHz, C_7_D_8_, 298 K): δ 2.46 (s, 6H, CMe), 2.25 (m, 6H, CH ^i^Pr), 1.16 (dvt, ^3^J_H–H_ = 7.1, N = 12.6, 36H, CH_3_^i^Pr_3_), −19.79 (t, ^2^J_H–P_ = 33.1, 2H, OsH_2_). ^31^P{^1^H} NMR (121.4 MHz, C_7_D_8_, 298 K): δ 48.9 (s; t under off-resonance conditions, ^2^J_C–P_ = 32.8). ^13^C{^1^H} APT NMR (75.48 MHz, C_7_D_8_, 298 K): δ 168.7 (t, ^2^J_C–P_ = 5.9, OsC), 30.3 (vt, N = 24.4, CH ^i^Pr), 20.8 (s, CH_3_^i^Pr), 20.1 (CMe). *T*_1(min)_ (ms, OsH, 300.13 MHz, C_7_D_8_, 203 K): 254 ± 5 (−19.79 ppm).

#### Preparation of OsH_2_(η^4^-H_2_CCHCHCH_2_)(P^i^Pr_3_)_2_ (**3**)

A solution of complex **2** (50 mg, 0.088 mmol) in toluene (1 mL) was heated at 80 °C for 24 h. Then, it was concentrated to dryness giving a white solid. Yield: 41.5 mg (83%). Colorless single crystals suitable for X-ray diffraction analysis were obtained from a saturated solution of **3** in pentane at −30 °C. Anal. Calcd. for C_22_H_50_OsP_2_: C, 46.62; H, 8.89. Found: C, 46.93; H, 9.21. HR-MS (electrospray): *m*/*z* calcd for C_22_H_49_OsP_2_ [M – H]^+^ 567.2919; found 567.2935. IR (ATR, cm^–1^): ν(Os–H) 2033. ^1^H NMR (300.13 MHz, C_7_D_8_, 298 K): δ 4.53 (br, 2H, HCCH), 2.07 (br, 2H, CH_2_), 1.96 (m, 6H, CH ^i^Pr), 1.09 (dvt, ^3^J_H–H_ = 5.6, N = 12.7, 36H, CH_3_^i^Pr_3_), −0.52 (br, 2H, CH_2_), −13.65 (t, ^2^J_H–P_ = 32.0, 2H, OsH_2_). ^31^P{^1^H} NMR (121.4 MHz, C_7_D_8_, 298 K): δ 37.9 (s). ^13^C{^1^H} APT NMR (75.48 MHz, C_7_D_8_, 298 K): δ 68.4 (HCCH), 30.7 (vt, N = 26.2, CH ^i^Pr), 25.5 (CH_2_), 20.0 (s, CH_3_^i^Pr). *T*_1(min)_ (ms, OsH, 400.13 MHz, C_7_D_8_, 203 K): 356 ± 5 (−13.65 ppm).

#### Preparation of **3**_**d**_**2**__

2-Butyne (6.0 μL, 0.077 mmol) was added to two NMR tubes containing deuterated complex **1**_**d**_**6**__ (20 mg, 0.039 mmol) in toluene-*d*_8_ (0.5 mL). The tubes were heated at 50 °C for 18 h. After this time, their ^1^H and ^31^P{^1^H} NMR spectra showed the quantitative transformation of **1**_**d**_**6**__ into OsD_2_(η^2^-C_2_Me_2_)(P^i^Pr_3_)_2_ (**2**_**d**_**2**__). Then, they were concentrated to dryness to give orange oils. The ^1^H NMR (300.13 MHz, toluene-d_8_, 298 K) data were identical to that reported for **2** except for the almost total disappearance of the signal at δ −19.79 (OsH). The two NMR tubes (A and B) with a solution of deuterated complex **2**_**d2**_ were heated at 80 °C for 24 h. Tetrachloroethane (8.2 μL, 0.078 mmol) was added to tube A as the internal standard. The ^1^H NMR (300.13 MHz, toluene-*d*_8_, 298 K) data were identical to those of **3** except for a decrease in the intensity of the signals δ 4.53 (77%, HCCH), −0.52 (76%, CH_2_), −13.65 (79%, OsH_2_). The percentages indicate the amount of hydrogen atoms in those positions. Tube B was concentrated to dryness to give a colorless oil, which was checked in nondeuterated toluene. ^2^H NMR (61.42 MHz, toluene, 298 K): δ 4.47 (br, DCCD), 2.02 (br, CD_2_), −0.57 (br, CD_2_), −13.66 (br, OsD_2_).

#### Reaction of **1** with 3-Hexyne: Formation of OsH_2_(η^2^-C_2_Et_2_)(P^i^Pr_3_)_2_ (**4**)

3-Hexyne (43 μL, 0.38 mmol) was added over a solution of complex **1** (100 mg, 0.19 mmol) in toluene (2 mL). The mixture was heated at 50 °C for 18 h. After this time, the ^1^H and ^31^P{^1^H} NMR spectra of the reaction crude shows a mixture of complexes **4** and **5** in an 88:22 ratio along with 1-hexene and 3-hexene. The mixture was concentrated to dryness giving an orange oil, which was washed with cold methanol (3 × 2 mL) and dried under vacuum. Anal. Calcd. for C_24_H_54_OsP_2_: C, 48.46; H, 9.15. Found: C, 48.10; H, 9.01. HR-MS (electrospray): *m*/*z* calcd for C_24_H_53_OsP_2_ [M – H]^+^ 595.3233; found 595.3255. ^1^H NMR (300.13 MHz, C_7_D_8_, 298 K): δ 3.08 (q, ^3^J_H–H_ = 7.4, 4H, CH_2_), 2.14 (m, 6H, CH ^i^Pr), 1.30 (t, ^3^J_H–H_ = 7.4, 6H, CH_3_ Et), 1.16 (dvt, ^3^J_H–H_ = 6.3, N = 12.6, 36H, CH_3_^i^Pr_3_), −18.14 (t, ^2^J_H–P_ = 33.1, 2H, OsH_2_). ^31^P{^1^H} NMR (121.4 MHz, C_7_D_8_, 298 K): δ 48.2 (s; t under off-resonance conditions, ^2^J_H–P_ = 33.1). ^13^C{^1^H} APT NMR (75.48 MHz, C_7_D_8_, 298 K): δ 176.4 (t, ^2^J_H–C_ = 6.3, OsC), 30.2 (s, CH_2_), 29.8 (vt, N = 23.6, CH ^i^Pr), 20.1 (s, CH_3_^i^Pr), 13.36 (s, CH_3_ Et).

#### Isomerization of **4**: Formation of OsH_2_(η^4^-H_2_CCHCHCHEt)(P^i^Pr_3_)_2_ (**5**) and OsH_2_(η^4^-MeHCCHCHCHMe)(P^i^Pr_3_)_2_ (**6**)

A mixture of complexes **4** and **5** in 88:22 molar ratio (30 mg, 0.05 mmol) was heated in toluene (0.5 mL) at 80 °C for 24 h. The mixture was concentrated to dryness obtaining a light-yellow oil. The oil was washed with cold pentane (2 × 1 mL) and dried under vacuo. ^1^H NMR spectra show a mixture of complexes **5** and **6** in a 9:1 molar ratio. Anal. Calcd. for C_24_H_54_OsP_2_: C, 48.46; H, 9.15. Found: C, 48.12; H, 8.88. HR-MS (electrospray): *m*/*z* calcd for C_24_H_53_OsP_2_ [M – H]^+^ 595.3233; found 595.3251. Spectroscopic data for **5**: ^1^H NMR (300.13 MHz, C_6_D_6_, 293 K): δ 4.42 (m, 1H, C*H*CHEt), 4.40 (m, 1H, CH_2_C*H*), 2.31 (m, 1H, CH_2_ Et), 2.12–1.95 (br, 7H, 1H C*H*_2_CH and 6H CH ^i^Pr_3_), 1.68 (m, 1H, CH_2_ Et), 1.26 (t, ^3^J_H–H_ = 7.4, 3H, CH_3_ Et), 1.12 (br, 36H, CH_3_^i^Pr_3_), 0.23 (br, 1H, C*H*Et), −0.32 (br, 1H, C*H*_2_CH), −13.46 (br, 1H, OsH), −14.14 (br, 1H, OsH). ^1^H NMR (300.13 MHz, C_7_D_8_, 253 K): δ 4.35 (m, 1H, C*H*CHEt), 4.33 (m, 1H, CH_2_C*H*), 2.35 (m, 1H, CH_2_ Et), 2.14 (br, 3H, CH ^i^Pr_3_), 1.83 (br, 4H, 1H C*H*_2_CH and 3 CH ^i^Pr_3_), 1.64 (m, 1H, CH_2_ Et), 1.29 (t, ^3^J_H–H_ = 7.3, 3H, CH_3_ Et), 1.20 (m, 18H, CH_3_^i^Pr_3_), 1.01 (m, 18H, CH_3_^i^Pr_3_), 0.14 (br, 1H, C*H*Et), −0.40 (br, 1H, C*H*_2_CH), −13.25 (dd, ^2^J_H–P_ = 27.3, 36.6, 1H, OsH), −14.40 (dd, ^2^J_H–P_ = 27.3, 36.6, 1H, OsH). ^31^P{^1^H} NMR (121.4 MHz, C_7_D_8_, 298 K): 28.7 (br AB system). ^31^P{^1^H} NMR (121.4 MHz, C_7_D_8_, 253 K): δ 28.7 (AB system, J_AB_ = 86.5 Hz; Δ*υ* = 979 Hz). ^13^C{^1^H} APT NMR (75.48 MHz, C_6_D_6_, 298 K): δ 73.2 (s, *C*HCHEt), 66.0 (s, CH_2_*C*H), 49.4 (s, *C*HEt), 31.5 (s, CH_2_ Et), 30.8 (br, CH ^i^Pr), 30.5 (br, CH ^i^Pr), 25.3 (s, *C*H_2_CH), 21.0 (s, CH_3_ Et), 20.2 (s, CH_3_^i^Pr), 20.1 (s, CH_3_^i^Pr). *T*_1(min)_ (ms, OsH, 400.13 MHz, C_7_D_8_, 213 K): 288 ± 5 (−18.36 ppm), 288 ± 5 (−19.50). Selected spectroscopic data for **6**: ^1^H NMR (300.13 MHz, C_6_D_6_, 298 K): δ 4.28 (m, 2H, C*H*CHMe), 1.90 (3H, CH*Me* overlapped with CH P^i^Pr_3_), 0.23 (inferred from COSY spectrum, 2H, C*H*Me), −14.11 (dd, ^2^J_H–P_ = 36.8, 28.2, 2H, OsH_2_). ^31^P{^1^H} NMR (121.4 MHz, C_7_D_8_, 298 K): δ 27.0 (AB system, J_AB_ = 89, Hz; Δ*υ* = 1145 Hz). ^13^C{^1^H} APT NMR (75.48 MHz, C_6_D_6_, 298 K): δ 71.0 (t, ^2^J_C–P_ = 3.4, *C*HCHMe), 39.3 (dd, ^2^J_C–P_ = 8.5, 6.1, *C*HMe), 21.7 (s, CH*Me*). *T*_1(min)_ (ms, OsH, 400.13 MHz, C_7_D_8_, 213 K): 309 ± 5 (−19.15 ppm).

#### Preparation of OsH{κ^2^-*H*,*H*-(H_2_Bpin)}(η^2^-HBpin)(P*^i^*Pr_3_)_2_ (**7**)

A solution of **2** (20 mg, 0.035 mmol) in toluene (0.5 mL) was treated with pinacolborane (25.4 μL, 0.175 mmol). The mixture was stirred at room temperature for 3 h (or 1 h at 60 °C), during which time the solution turned colorless and the ^1^H NMR spectrum of reaction crude in toluene-*d*_8_ showed the formation of **2** along with 1-butene. The reaction crude was concentrated to dryness affording a white solid. The solid was washed with cold pentane (0.5 mL, −72 °C) and dried under vacuum. Yield: 19 mg (71%). Colorless single crystals suitable for X-ray diffraction analysis were obtained by removing the solvent of a saturated solution of **7** in toluene at room temperature under vacuum. Anal. Calcd. for C_30_H_70_B_2_O_4_OsP_2_: C, 46.88; H, 9.18. Found: C, 47.27; H, 8.84. ^1^H NMR (300.13 MHz, C_7_D_8_, 298 K): δ 2.43 (m, 6H, CH ^i^Pr_3_), 1.28 (dvt, ^3^J_H–H_ = 5.7, N = 12.8, 36H, CH_3_^i^Pr_3_), 1.14 (s, 24H, CH_3_ Bpin), −10.65 (br, 4H, OsH_4_). ^1^H NMR (300.13 MHz, C_7_D_8_, 193 K): δ 2.43 (br, 6H, CH ^i^Pr_3_), 1.28 (br, 36H, CH_3_^i^Pr_3_), 1.14 (s, 24H, CH_3_ Bpin), −9.44 (br, 1H, OsH), −9.64 (br t, 1H, OsH), −11.88 (br, 2H, OsH_2_).^31^P{^1^H} NMR (121.4 MHz, C_7_D_8_, 298 K): δ 33.7. ^11^B{^1^H} NMR (94.29 MHz, C_7_D_8_, 298 K): δ 37.9. T_1(min)_ (ms, OsH, 400.13 MHz, C_7_D_8_, 273 K): 313 ± 5 (OsH_4_, −10.72 ppm).

#### Reaction of **7** with H_2_

A solution of complex **7** (10 mg, 0.013 mmol) in toluene-*d*_8_ (0.5 mL) was kept for 3 h under H_2_ atmosphere at room temperature. After that time, the ^1^H and ^31^P NMR spectra of the reaction mixture showed the quantitative transformation of **7** into OsH_3_(κ^2^-*H*,*H*-H_2_Bpin)(P^i^Pr_3_)_2_ (**8**).

#### Reaction of **7** with 2-Butyne

2-Butyne (7 μL, 0.195 mmol) was added to a solution of **7** (30 mg, 0.039 mmol) in 0.5 mL of toluene-*d*_8_ at room temperature, and the mixture was stirred for 30 min. After this time, the ^31^P NMR showed the quantitative transformation of **7** into **2**, whereas the ^1^H NMR showed also the formation of 2-pinacolboryl-1-butene.

#### Hydroboration of 2-Butyne and 3-Hexyne

The corresponding alkyne (0.18 mmol) was added over a solution of complex **2** (5 mg, 9.0 × 10^–3^ mmol) or complex **1** (4.7 mg, 9.0 × 10^–3^ mmol), pinacolborane (39 μL, 0.27 mmol), and mesytilene (25 μL, 0.18 mmol) as internal standard in toluene-*d*_8_ (0.5 mL). The mixture was heated at 60 °C and followed by ^1^H NMR spectra until the complete disappearance of the alkyne.

#### Isolation of Borylated Olefins

The corresponding alkyne (0.72 mmol) was added over a solution of complex **2** (20 mg, 0.036 mmol) and pinacolborane (156 μL, 1.08 mmol) in toluene (2 mL). The mixture was heated at 60 °C, 2 h for 2-butyne and 3 h for 3-hexyne. The solvent was evaporated to dryness under vacuum giving orange oils, which were purified by silica-gel flash chromatography using hexane as eluent. 2-Pinacolboryl-1-butene was isolated as a colorless oil in 82% yield, and 2-(hex-5-en-3-yl)-pinacolborane was isolated as a colorless oil in 77% yield.

#### Spectroscopic Data for 2-Pinacolboryl-1-butene

^1^H NMR (300.13 MHz, CDCl_3_, 298 K): δ 5.76 (d, 1H, ^3^J_H–H_ = 3.4, =CH_2_), 5.62 (br, 1H, =CH_2_), 2.18 (q, 2H, ^3^J_H–H_ = 7.5, CH_2_), 1.28 (s, 3H, CH_3_ Bpin), 1.03 (t, 3H, ^3^J_H–H_ = 7.5, CH_3_). ^13^C{^1^H} APT NMR (75.48 MHz, CDCl_3_, 298 K): δ127.8 (=CH_2_), 83.3 (C_q_ Bpin), 28.2 (CH_2_), 24.7 (CH_3_ Bpin), 13.6 (CH_3_), (CBpin, not observed). ^11^B NMR (96.29 MHz, CDCl_3_, 298 K): δ 30.0 (Bpin). These spectroscopic data agree with the reported data.^42^

#### Spectroscopic Data for 4-Pinacolboryl-1-hexene

^1^H NMR (300.13 MHz, CDCl_3_, 298 K): δ 5.74 (ddt, ^3^J_H–H_ = 17.0, ^3^J_H–H_ = 10.1, ^3^J_H–H_ = 6.9, 1H, CH_2_=C*H*), 4.94 (ddt, ^3^J_H–H_ = 17.0, ^3^J_H–H_ = 2.1, ^4^J_H–H_ = 1.5, 1H, CH=C*H*_2_), 4.86 (ddt, ^3^J_H–H_ = 10.1, ^3^J_H–H_ = 2.1, ^4^J_H–H_ = 1.0, 1H, CH=C*H*_2_), 2.07 (m, 3H, =CHC*H*_2_), 1.34 (m, 3H, C*H*BpinCH_2_C*H*_3_), 1.17 (s, 12H, CH_3_ Bpin), 0.84 (t, ^3^J_H–H_ = 6.9, 3H, CH_2_C*H*_3_). ^13^C{^1^H} APT NMR (75.48 MHz, CDCl_3_, 298 K): δ 138.7 (−*C*H=CH_2_), 114.6 (−CH=*C*H_2_), 82.8 (C_q_ Bpin), 35.3 (=CH*C*H_2_), 24.8 (CH_3_ Bpin), 23.8 (*C*H_2_CH_3_), 13.5 (CH_2_C*H*_3_). ^11^B NMR (96.29 MHz, CDCl_3_, 298 K): δ 34.4 (Bpin). These spectroscopic data are in agreement with those reported for the closely related 4-pinacolboryl-1-nonene and 4-pinacolboryl-1-pentene.^43^

## Supporting Information Available

General information, crystallographic data, computational details, and NMR spectra (PDF)
Cartesian coordinates of calculated structures (XYZ)

## Supplementary Material

om2c00338_si_001.pdf

om2c00338_si_002.xyz
