# Supplementary material for: Reactions of an Osmium–Hexahydride Complex with 2-Butyne and 3-Hexyne and Their Performance in the Migratory Hydroboration of Aliphatic Internal Alkynes
Source: Organometallics. 2022 Aug 31;41(17):2513–24. doi: 10.1021/acs.organomet.2c00338 (PMC9969483; doi:10.1021/acs.organomet.2c00338)
Supplement: Supplementary file 1 — om2c00338_si_001.pdf [file om2c00338_si_001.pdf]

Supporting Information

Reactions of an Osmium-Hexahydride Complex with 2-Butyne and 3-Hexyne and their Performance in the Migratory Hydroboration of Aliphatic Internal Alkynes

Juan C. Babón, Miguel A. Esteruelas, Ana M. López, and Enrique Oñate*

Departamento de Química Inorgánica, Instituto de Síntesis Química y Catálisis Homogénea (ISQCH), Centro de Innovación en Química Avanzada (ORFEO-CINQA), Universidad de Zaragoza-CSIC, 50009 Zaragoza, Spain

* Corresponding author's e-mail address: maester@unizar.es

Contents:

Experimental Section: General Information... S2

Structural Analysis of Complexes **2**, **3**, and **7**... S2

Computational Details S3

Energies of Optimized Structures S4

NMR spectra of complexes **2-7**... S5

^{1}H NMR spectra of the reaction crude of the catalytic hydroborations, and ^{1}H, ^{13}C{^{1}H} APT, and ^{11}B{^{1}H} NMR of 2-pinacolboryl-1-butene and 4-pinacolboryl-1-hexene.. S19

References.. S23

Experimental Section: General information. All manipulations were performed with rigorous exclusion of air at an argon/vacuum manifold using standard Schlenk-tube or glovebox techniques. Solvents were dried by the usual procedures and distilled under argon prior to use or obtained oxygen- and water-free from an MBraun solvent purification apparatus. Toluene-d_8 was stored over sodium in the glovebox. Complex $OsH_6(P^iPr_3)_2$ (**1**)[1] and $OsD_6(P^iPr_3)_2$ (**1$_{d6}$**)[2] were prepared according to the published methods. Alkynes were purchased from commercial sources. 2-Butyne was used without further purification whereas 3-hexyne was distilled in a Kugelrohr distillation oven. Pinacolborane was purchased from commercial sources and used without further purification. NMR spectra were recorded on a Bruker ARX 300, Bruker Avance 300 MHz, or a Bruker Avance 400 MHz instruments. Chemical shifts (expressed in parts per million) are referenced to residual solvent peaks (^1H, $^{13}C\{^1H\}$) and external H_3PO_4 ($^{31}P\{^1H\}$) or $BF_3{\cdot}OEt_2$ (^{11}B). Coupling constants J and N ($N = {}^3J_{H\text{-}P} + {}^5J_{H\text{-}P'}$ for ^1H or ${}^1J_{C\text{-}P} + {}^3J_{C\text{-}P'}$ for ^{13}C) are given in Hertz. High-resolution (HRMS) electrospray mass spectra were acquired using a MicroTOF-Q hybrid quadrupole time-of-flight spectrometer (Bruker Daltonics, Bremen, Germany). C, H, and N analyses were carried out in a Perkin-Elmer 2400-B Series II CHNS-Analyzer. Attenuated total reflection infrared spectra (ATR-IR) of solid samples were run on a Perkin-Elmer Spectrum 100 FT-IR spectrometer.

Structural Analysis of complexes 2, 3, and 7. X-ray data were collected on a APEX D8 Venture Bruker diffractometer (Mo radiation, $\lambda = 0.71073$ Å). The crystals were cooled with a nitrogen flow from Oxford Cryosystems system. Data were corrected for absorption by using a multiscan method applied with the SADABS program.[3] The structures were solved by Patterson or direct methods and refined by full-matrix least squares on F^2 with SHELXL2016 or SHELXL2019,[4] including isotropic and subsequently anisotropic displacement parameters. The hydrogen atoms were observed in the last Fourier Maps or calculated, and refined freely or using a restricted riding model.

Complexes **2** and **3** crystallize in the monoclinic C2/c space group. The asymmetric unit is made up of half a molecule, since a binary axis divides it in two. **7** crystallizes in a monoclinic P2/c space group which $\beta \simeq 90°$ given rise to a pseudomerohedral twin. The crystal was solved and refined in the monoclinic P2/c space group with the twin matrix (1 0 0 0 -1 0 0 0 -1) with BASF 0.07661. The asymmetric unit consists of a complete molecule and two halves. The two halves are the result of a binary axis that crosses the

molecules at the metallic centre, creating disorder in the position of the hydrides and making the boryl and borohydride ligands equal. In the discussion of results, reference is made only to the non-disordered molecule, whose structure has been validated by theoretical methods at the DFT level.

Crystal data for **2** (CCDC 2184027): $C_{22}H_{50}OsP_2$, Mw 566.76, orange, irregular block (0.150 x 0.140 x 0.100 mm^3), monoclinic, space group C2/c, *a*: 12.1297(7) Å, *b*: 10.3845(6) Å, *c*: 20.5139(13) Å, *β*: 103.1102(17)°, *V* = 2516.6(3) Å^3, *Z* = 4, *Z'* = 0.5, D_{calc}: 1.496 g cm^{-3}, F(000): 1152, *T* = 100(2) K, μ 5.198 mm^{-1}. 43369 measured reflections (2θ: 3-57°, ω and φ scans 0.5°), 3132 unique (R_{int} = 0.0669); min./max. transm. Factors 0.677/0.862. Final agreement factors were R^1 = 0.0239 (2901 observed reflections, I > 2σ(I)) and wR^2 = 0.0543; data/restraints/parameters 3132/1/124; GoF = 0.985. Largest peak and hole 5.874 (close to Os atoms) and -1.982 e/ Å^3.

Crystal data for **3** (CCDC 2184026): $C_{22}H_{50}OsP_2$, Mw 566.76, pale yellow, irregular block (0.160 x 0.150 x 0.100 mm^3), monoclinic, space group C2/c, *a*: 8.6075(4) Å, *b*: 15.3758(6)Å, *c*: 18.7582(8) Å, *β*: 92.0630(15)°, *V* = 2480.99(18) Å^3, *Z* = 4, *Z'* = 0.5, D_{calc}: 1.517 g cm^{-3}, F(000): 1152, *T* = 100(2) K, μ 5.273 mm^{-1}. 43647 measured reflections (2θ: 3-57°, ω and φ scans 0.5°), 3005 unique (R_{int} = 0.0345); min./max. transm. Factors 0.778/0.862. Final agreement factors were R^1 = 0.0498 (2963 observed reflections, I > 2σ(I)) and wR^2 = 0.1270; data/restraints/parameters 3005 /14/168; GoF = 1.433. Largest peak and hole 2.232 (close to Os atoms) and -1.883 e/ Å^3.

Crystal data for **7** (CCDC 2184028): $C_{30}H_{70}B_2O_4OsP_2$, Mw 768.62, colorless, irregular block (0.145 x 0.087 x 0.026 mm^3), monoclinic, space group P2/c, *a*: 21.6740(8) Å, *b*: 9.3227(4) Å, *c*: 37.1226(15) Å, *β*: 90.708(2)°, *V* = 7500.4(5) Å^3, *Z* = 8, *Z'* = 1+0.5+0.5, D_{calc}: 1.361 g cm^{-3}, F(000): 3184, *T* = 100(2) K, μ 3.515 mm^{-1}. 236450 measured reflections (2θ: 3-57°, ω and φ scans 0.5°), 18653 unique (R_{int} = 0.0591); min./max. transm. Factors 0.730/0.862. Final agreement factors were R^1 = 0.0630 (17808 observed reflections, I > 2σ(I)) and wR^2 = 0.1411; data/restraints/parameters 18653 /28/766; GoF = 1.349. Largest peak and hole 3.929 (close to Os atoms) and -8.110 e/ Å^3.

Computational Details. The optimization of **2** and **3** were performed at the DFT level using the B3LYP functional[5] supplemented with the Grimme's dispersion correction D3[6] as implemented in Gaussian09.[7] Os atoms were described by means of an effective core potential SDD for the inner electron[8] and its associated double-ζ basis set for the outer

ones, complemented with a set of f-polarization functions for osmium.[9] The 6-31G** basis set was used for all the other atoms.[10] This level is denoted B3LYP-D3//SDD(f)/631g**.

Geometry optimization for **7** were performed without symmetry constraints using the Gaussian09[7] suite of programs at the BP86[11] /def2-TZVPP[12] level of theory using the D3 dispersion correction.[6] This level is denoted BP86-D3/def2-TZVPP.

All species were characterized by frequency calculations, and have positive definite Hessian matrices thus confirming that the computed structures are minima on the potential energy surface. The topology of the electron density was conducted using the AIMAll program package.[13]

Energies of the optimized structures

2

Zero-point correction= 0.678066 (Hartree/Particle)
Thermal correction to Energy= 0.715977
Thermal correction to Enthalpy= 0.716921
Thermal correction to Gibbs Free Energy= 0.611070
Sum of electronic and zero-point Energies= -1641.444583
Sum of electronic and thermal Energies= -1641.406672
Sum of electronic and thermal Enthalpies= -1641.405728
Sum of electronic and thermal Free Energies= -1641.511579

3

Zero-point correction= 0.682693 (Hartree/Particle)
Thermal correction to Energy= 0.718190
Thermal correction to Enthalpy= 0.719134
Thermal correction to Gibbs Free Energy= 0.619853
Sum of electronic and zero-point Energies= -1641.440223
Sum of electronic and thermal Energies= -1641.404727
Sum of electronic and thermal Enthalpies= -1641.403783
Sum of electronic and thermal Free Energies= -1641.503064

7- (tz):

Zero-point correction= 0.943106 (Hartree/Particle)
Thermal correction to Energy= 0.997219
Thermal correction to Enthalpy= 0.998164
Thermal correction to Gibbs Free Energy= 0.860875
Sum of electronic and zero-point Energies= -2309.727457
Sum of electronic and thermal Energies= -2309.673344
Sum of electronic and thermal Enthalpies= -2309.672399
Sum of electronic and thermal Free Energies= -2309.809689

NMR spectra of complexes 2-7

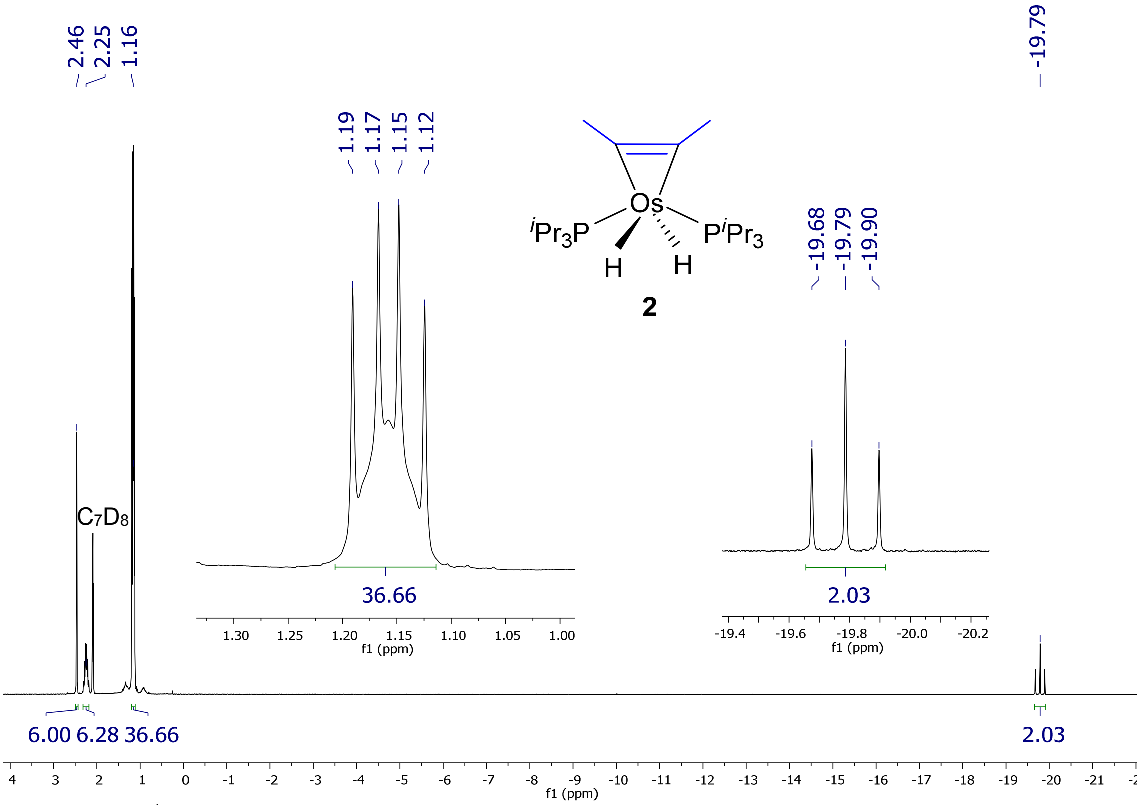

Figure S1. ^1H NMR (300.13 MHz, C_7D_8, 298 K) spectrum of complex **2**.

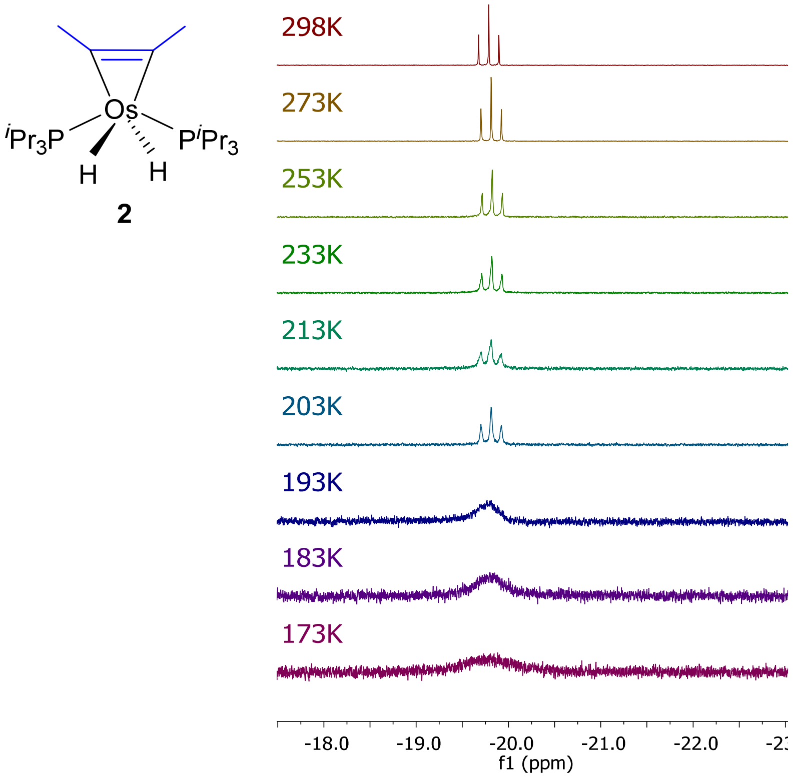

Figure S2. High-field region of the ^1H NMR (300.13 MHz, C_7D_8) spectrum of complex **2** between 298 and 173 K.

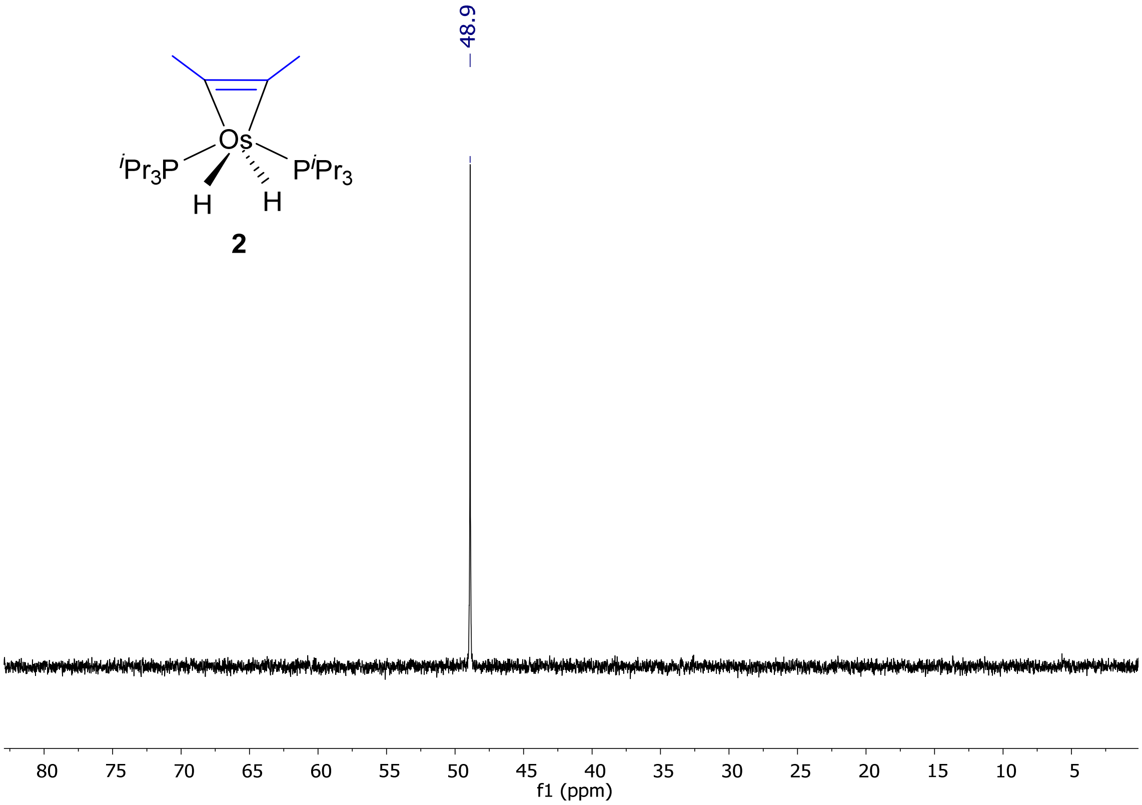

Figure S3. $^{31}P\ \{^1H\}$ NMR (121.50 MHz, C_7D_8, 298 K) spectrum of complex **2**.

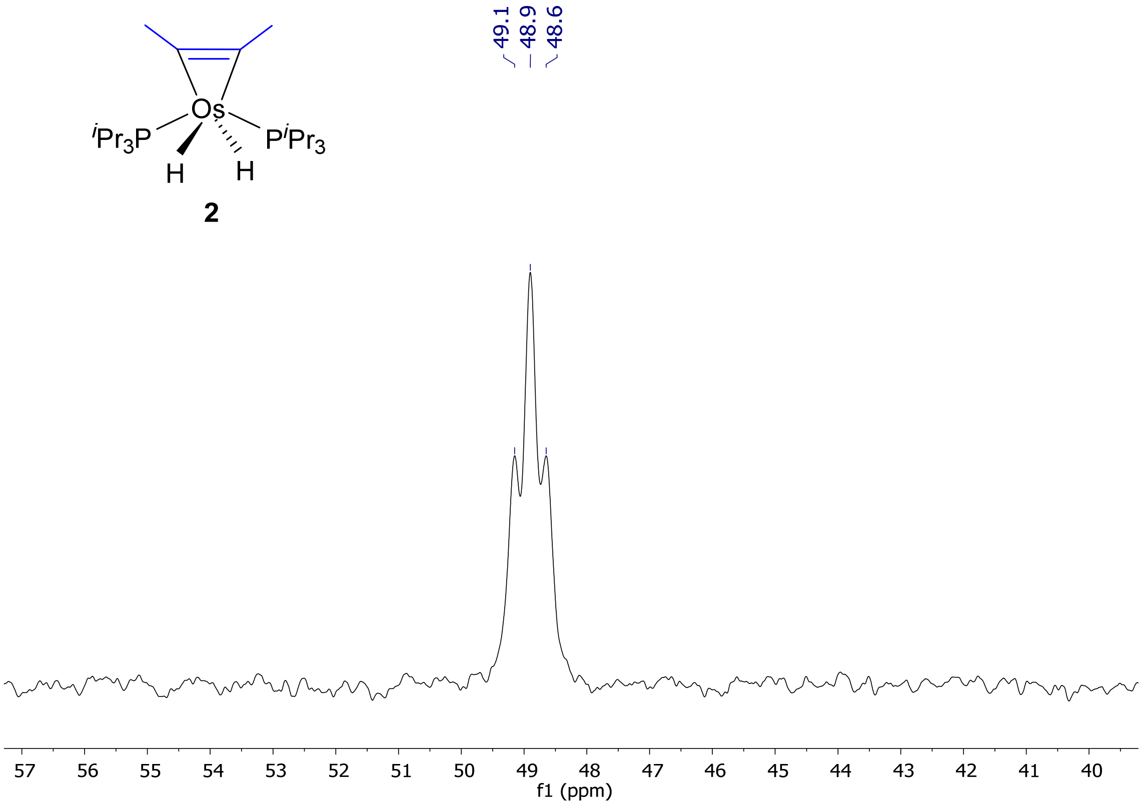

Figure S4. Off-resonance ^{31}P NMR (121.50 MHz, C_7D_8, 298 K) spectrum of complex **2** showing the selective coupling to the hydrides.

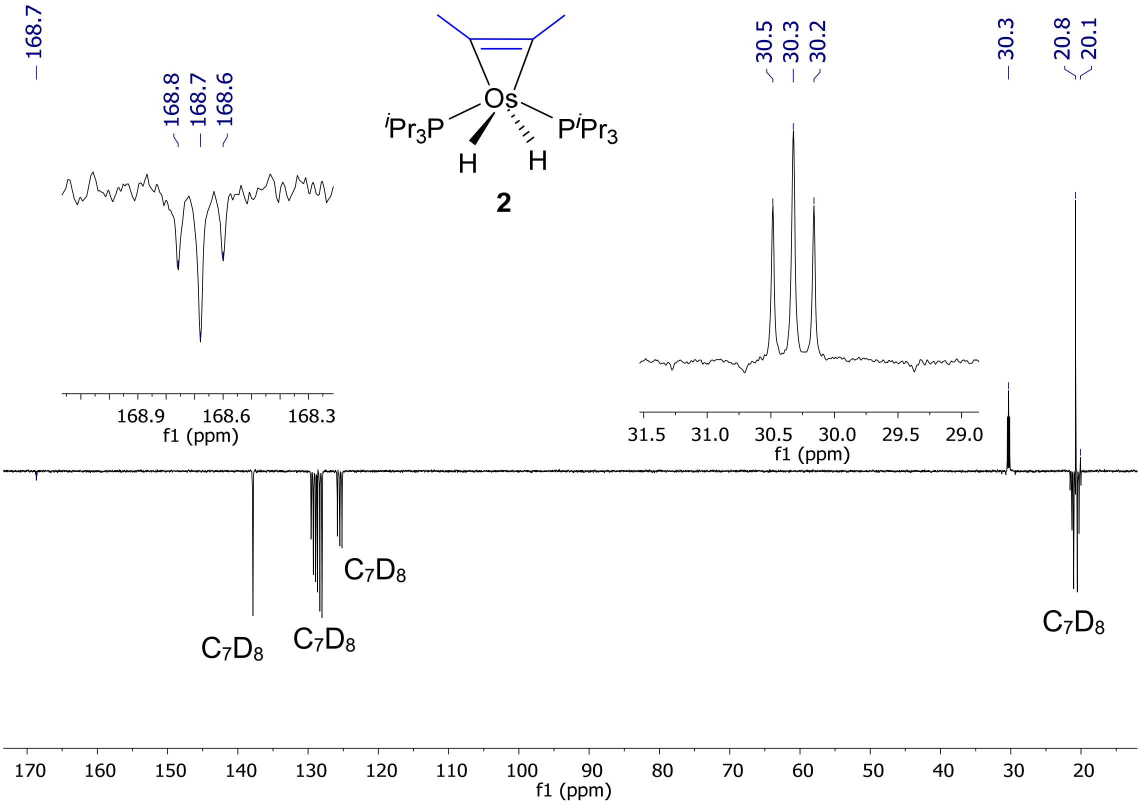

Figure S5. $^{13}C\{^{1}H\}$ APT NMR (75.48 MHz, C_7D_8, 298 K) spectrum of complex **2**.

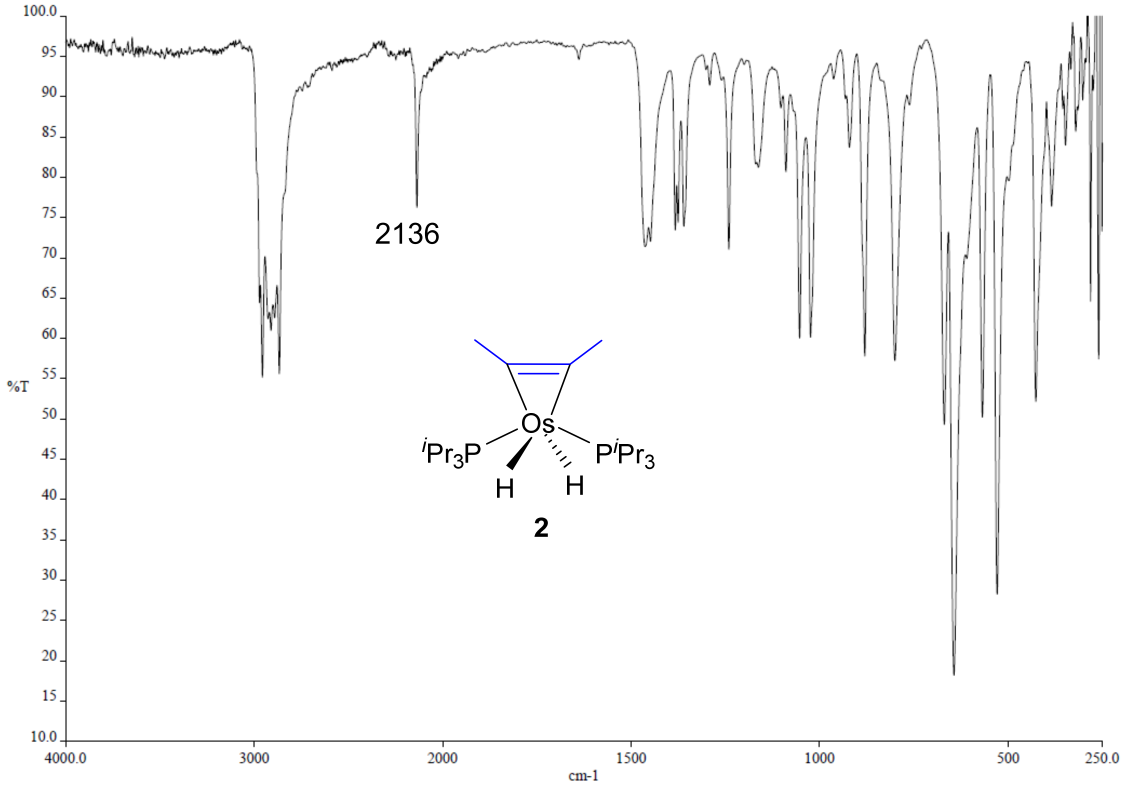

Figure S6. IR ATR spectrum of complex **2**.

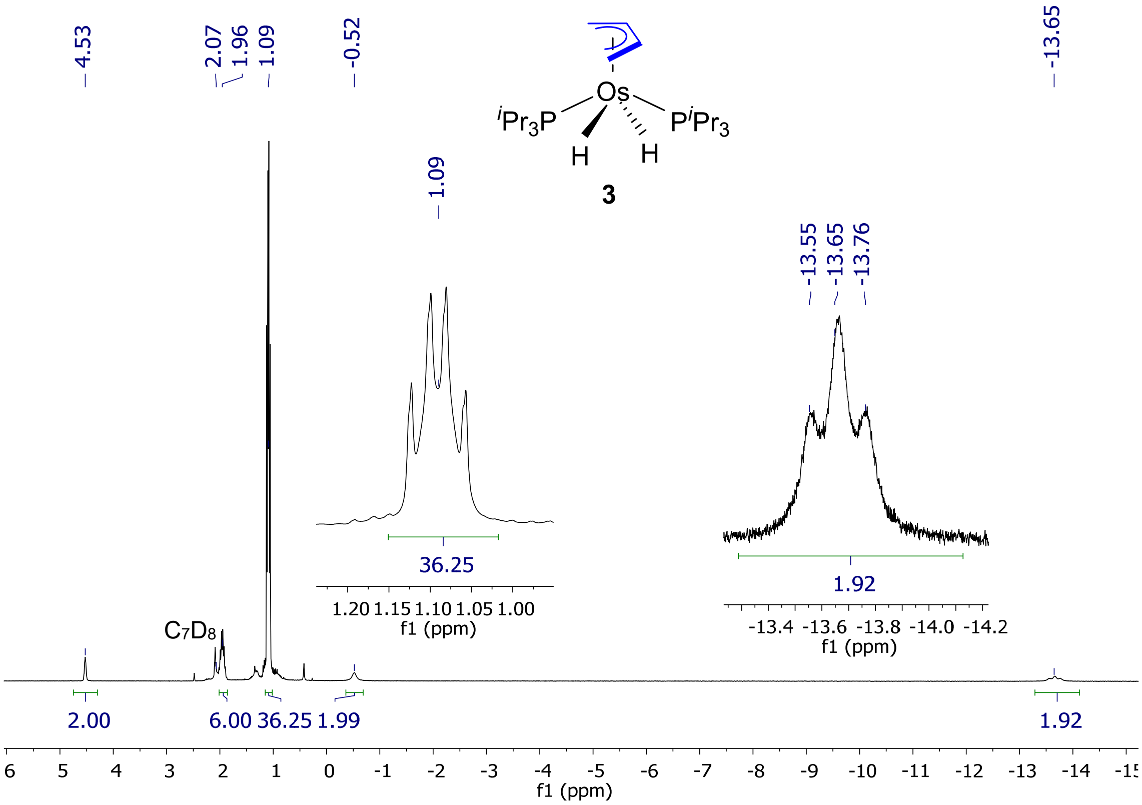

Figure S7. ^{1}H NMR (300.13 MHz, C_7D_8, 298 K) spectrum of complex **3**.

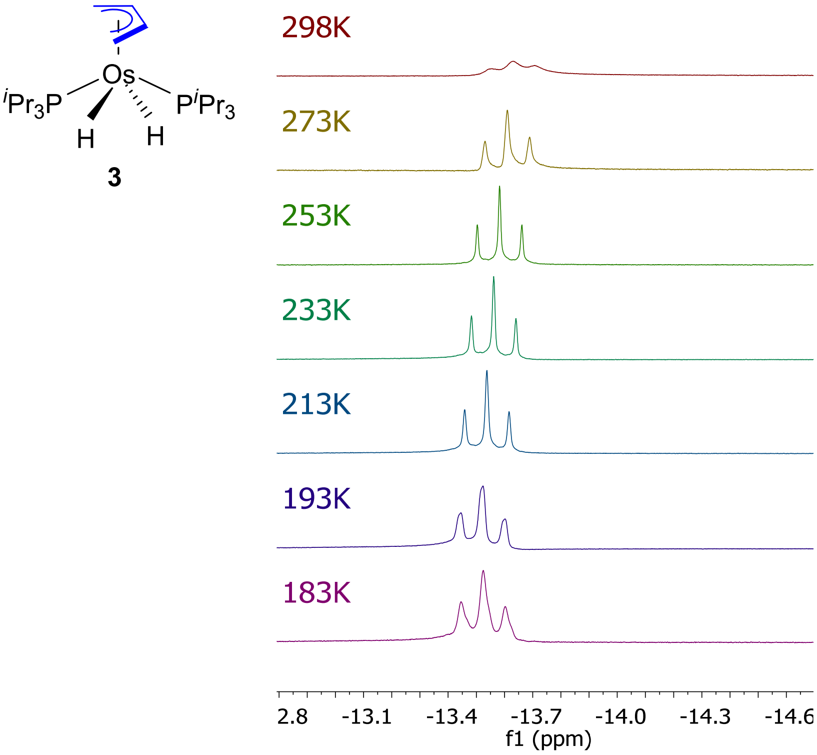

Figure S8. High-field region of the ^{1}H NMR (400.13 MHz, C_7D_8) spectrum of complex **3** between 298 and 183 K.

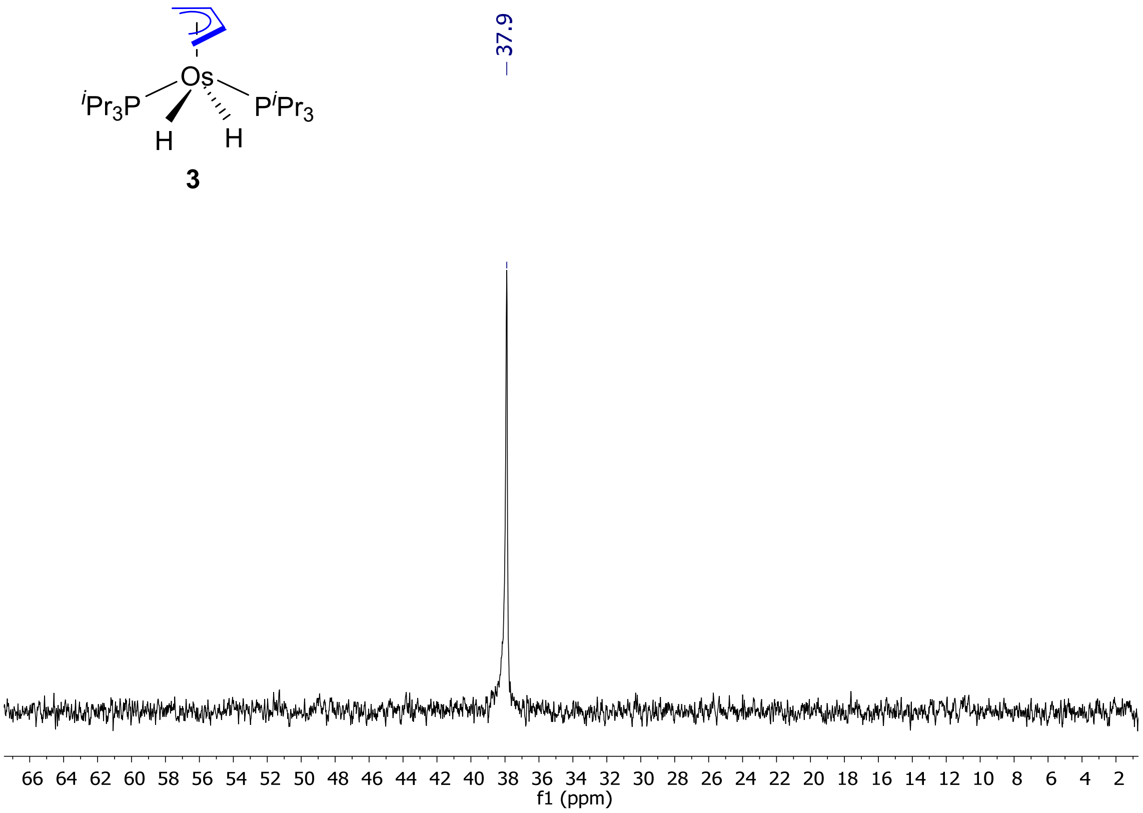

Figure S9. ^{31}P {^{1}H} NMR (121.50 MHz, C_7D_8, 298 K) spectrum of complex **3**.

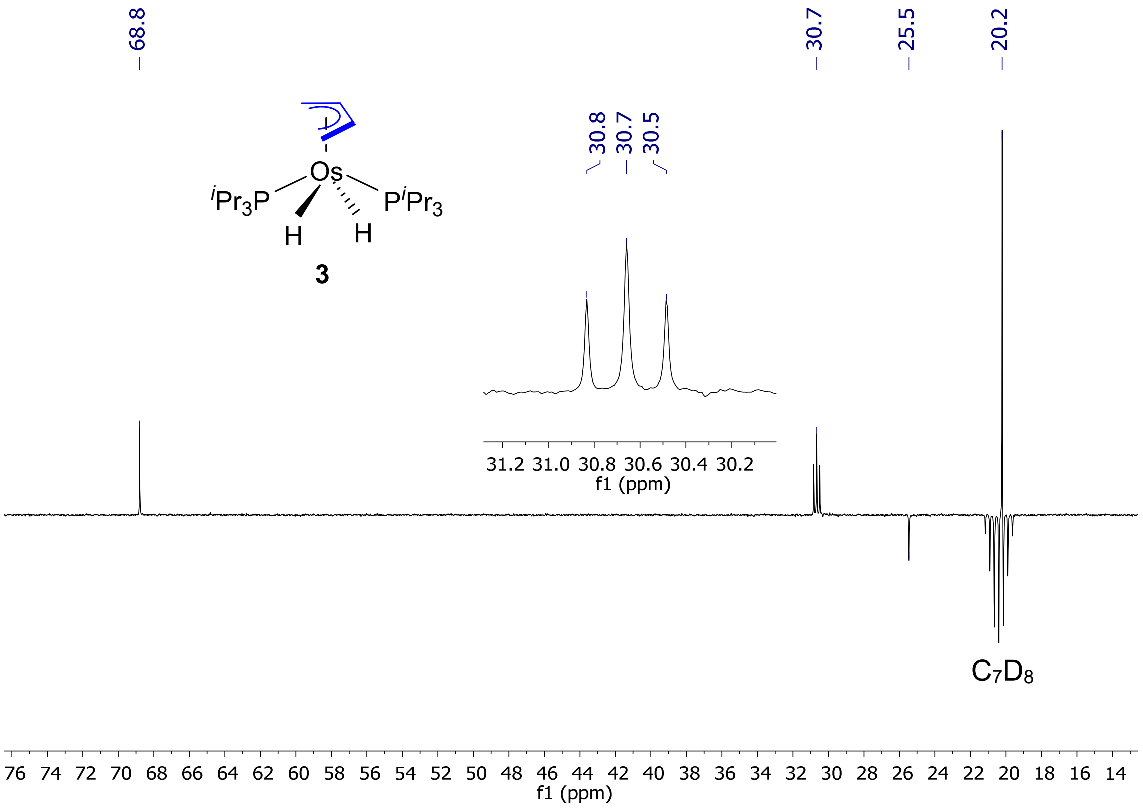

Figure S10. ^{13}C{^{1}H} APT NMR (75.48 MHz, C_7D_8, 298 K) spectrum of complex **3**.

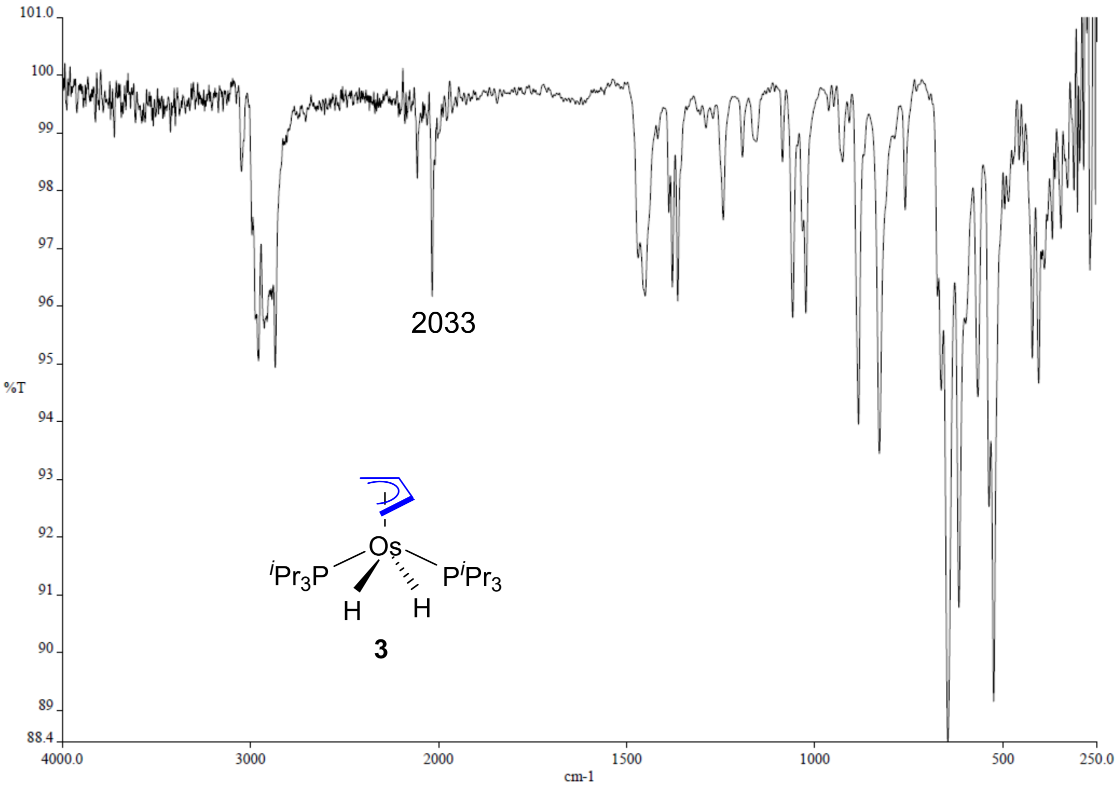

Figure S11. IR ATR spectrum of complex **3**.

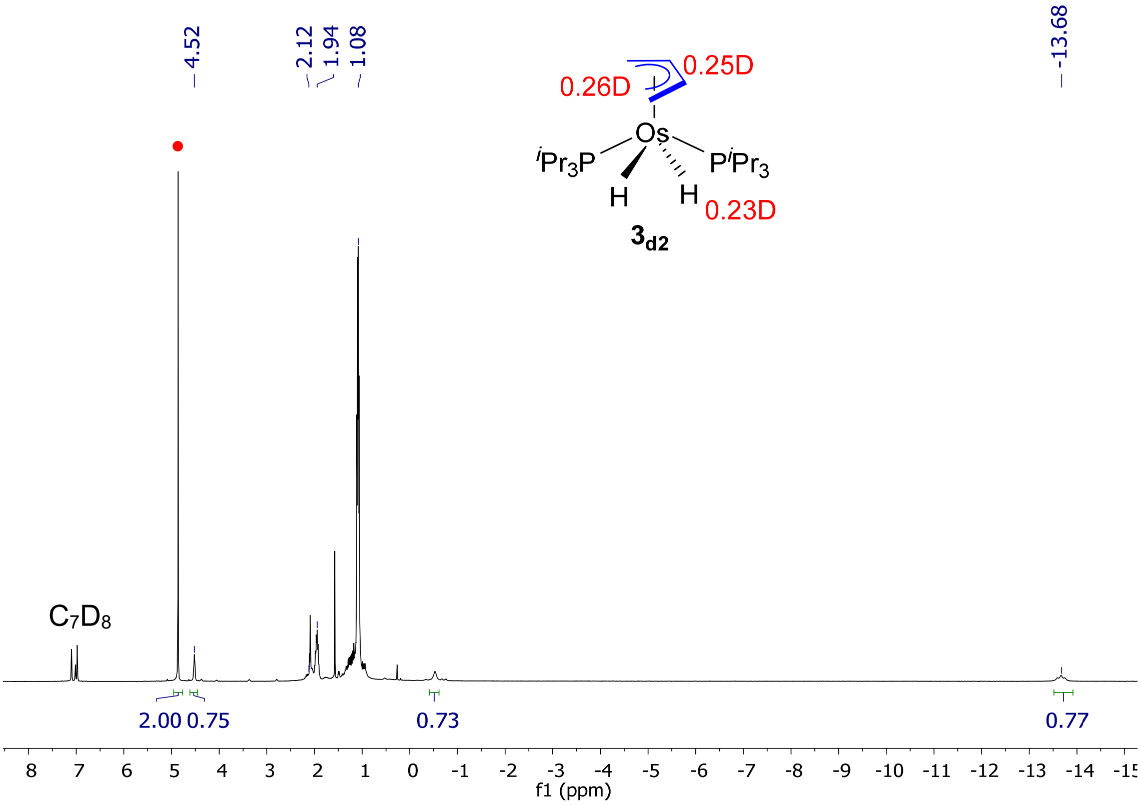

Figure S12. ^1H NMR (400.16 MHz, C_7D_8, 298 K) spectrum of complex $\mathbf{3_{d2}}$. Internal standard 1,1,2,2-tetrachloroethane (●).

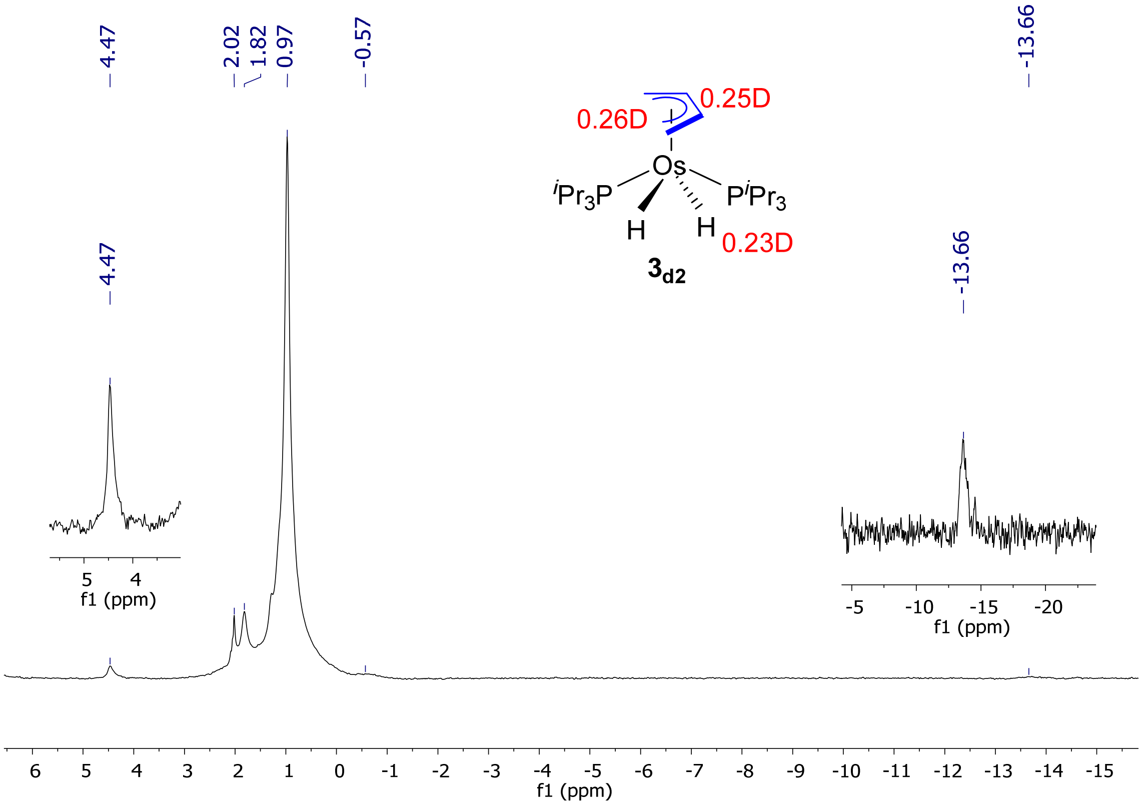

Figure S13. ^{2}H NMR (61.42 MHz, Toluene, 298 K) spectrum of complex **3$_{d2}$**.

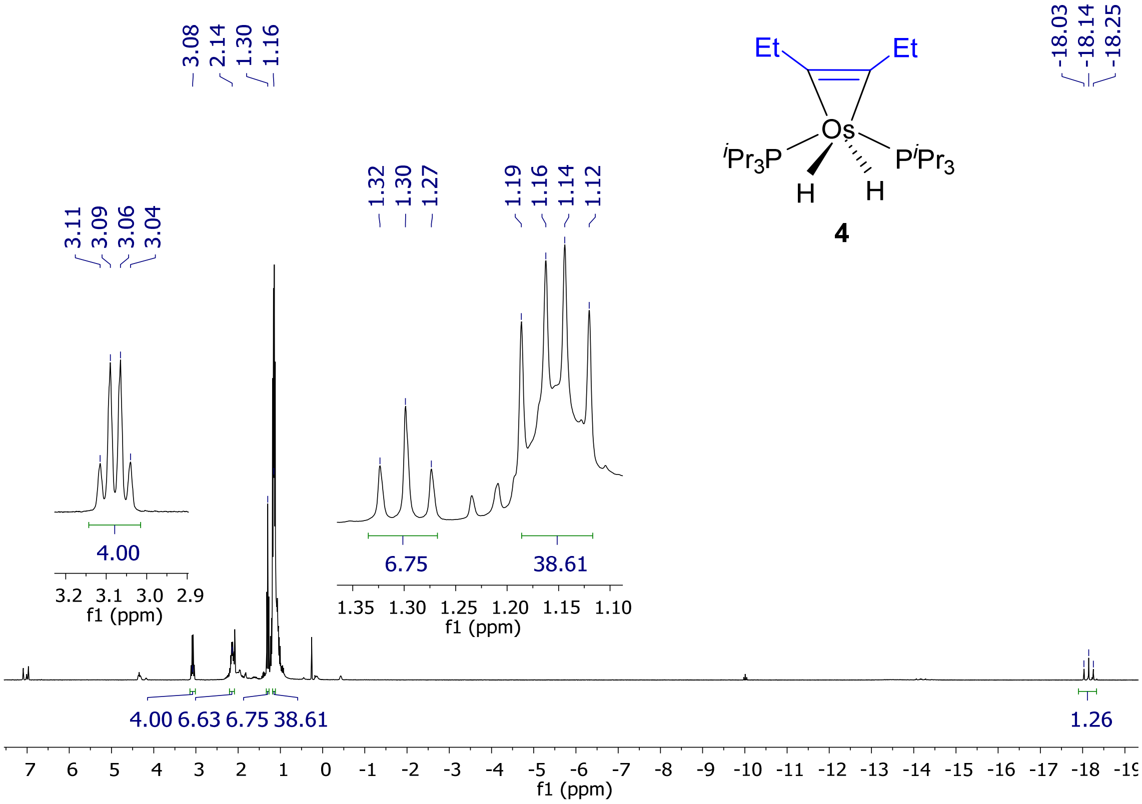

Figure S14. ^{1}H NMR (300.13 MHz, C_7D_8, 298 K) spectrum of complex **4**.

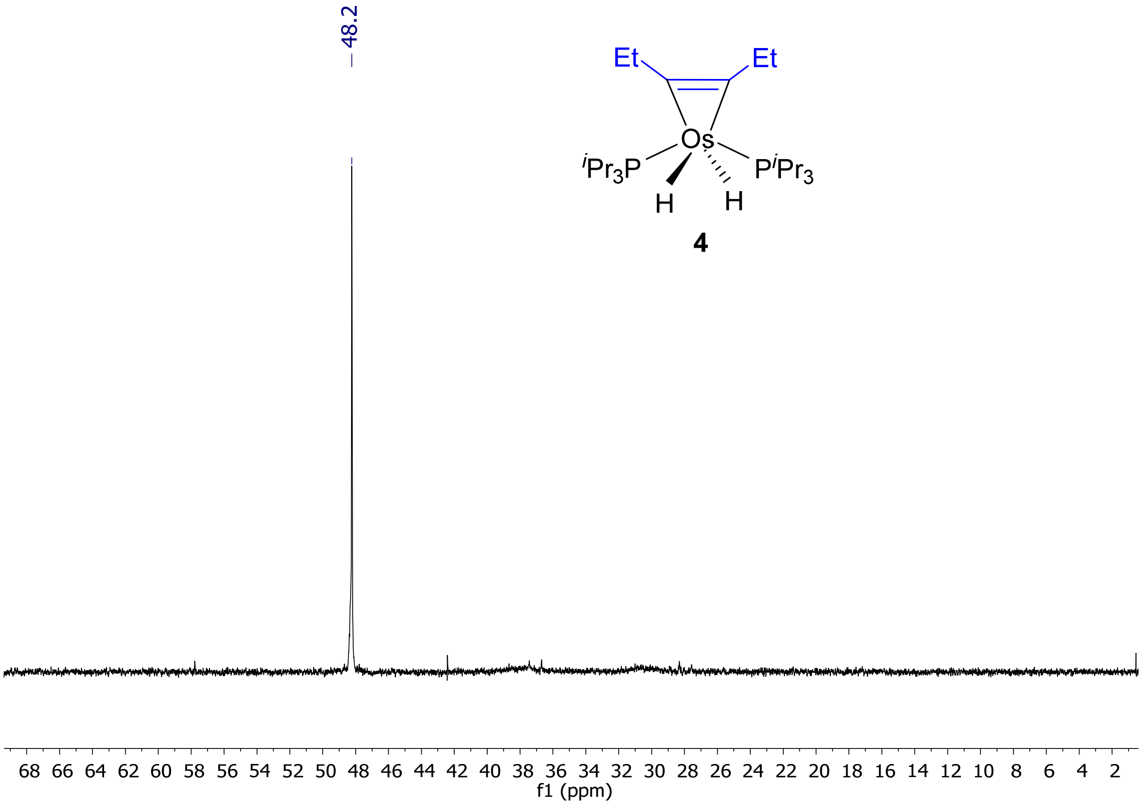

Figure S15. $^{31}P\ \{^{1}H\}$ NMR (121.50 MHz, C_7D_8, 298 K) spectrum of complex **4**.

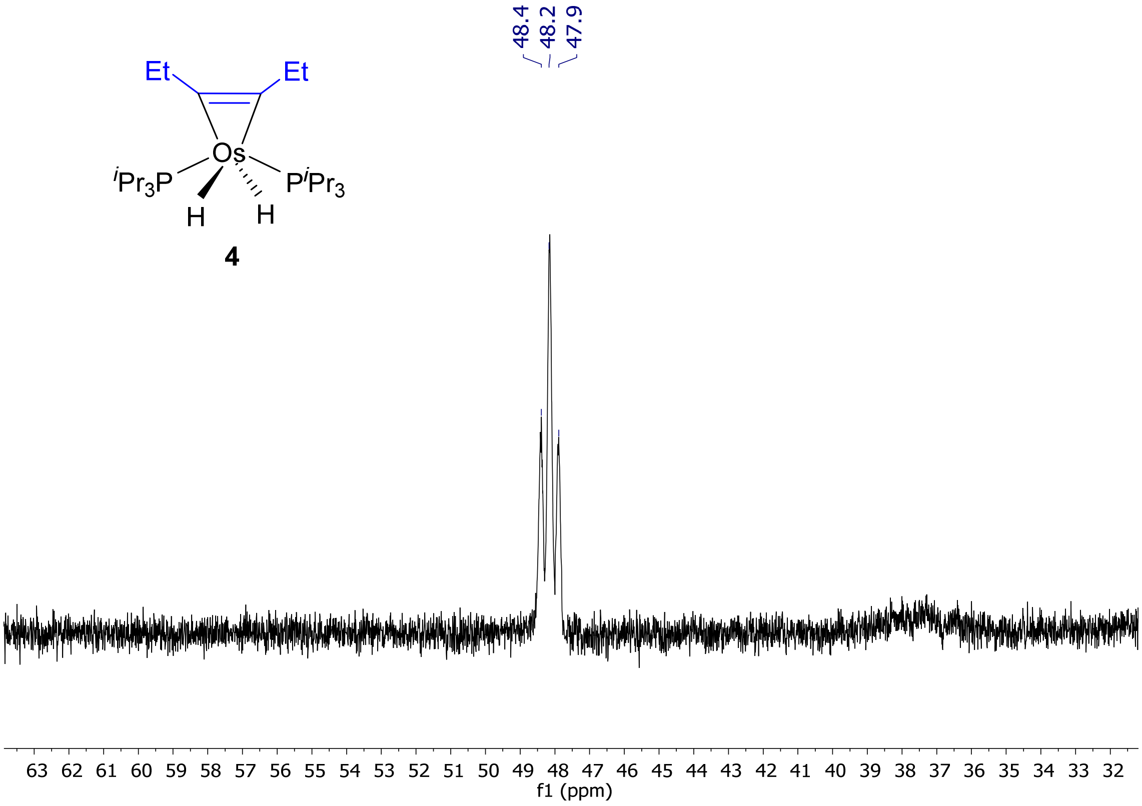

Figure S16. Off-resonance ^{31}P NMR (121.50 MHz, C_7D_8, 298 K) spectrum of complex **4** showing the selective coupling to the hydrides.

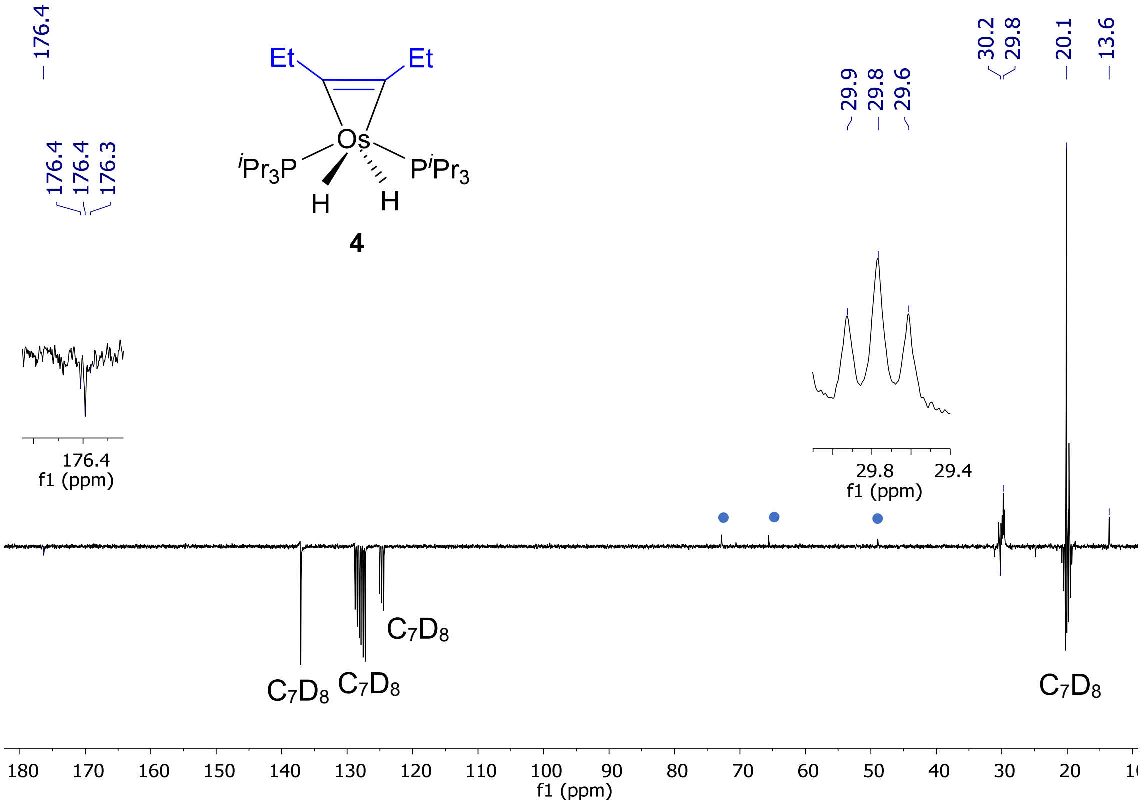

Figure S17. $^{13}C\{^1H\}$ APT NMR (75.48 MHz, C_7D_8, 298 K) spectrum of complex **4**. Complex **5** (•).

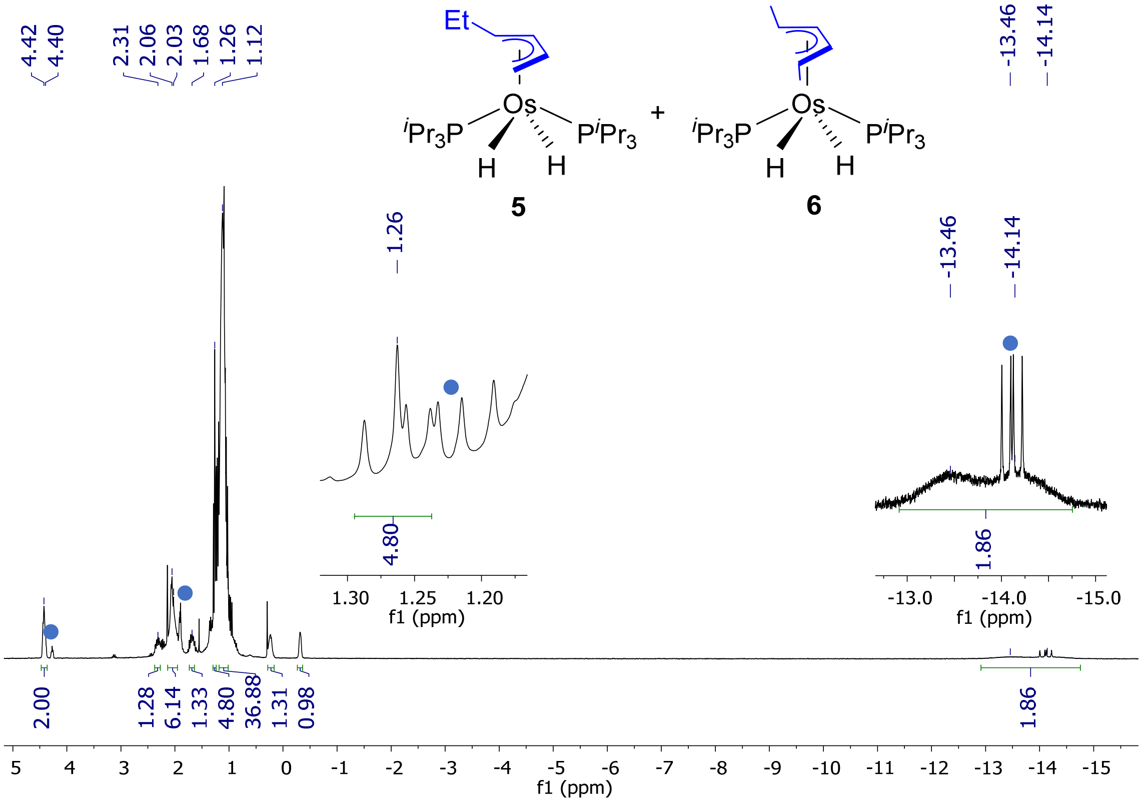

Figure S18. ^1H NMR (300.13 MHz, C_6D_6, 298 K) spectrum of complexes **5** and **6** (•).

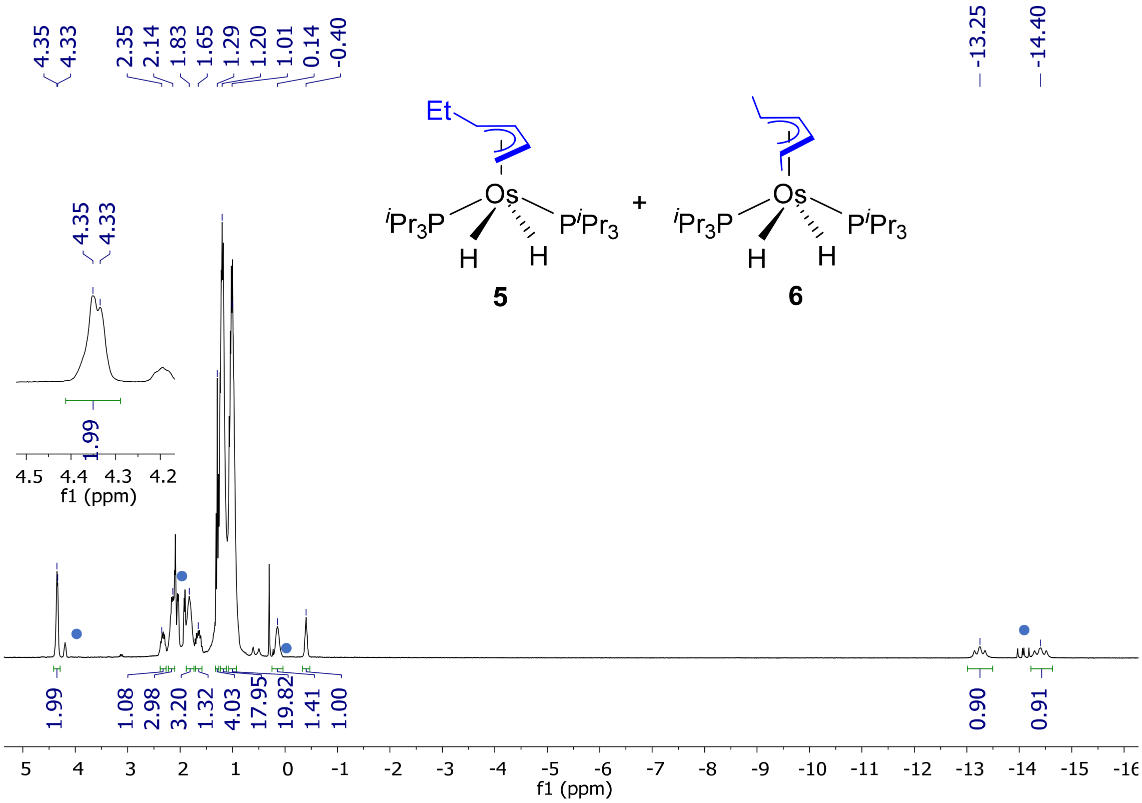

Figure S19. ^{1}H NMR (300.13 MHz, C_7D_8, 253 K) spectrum of complexes **5** and **6** (●).

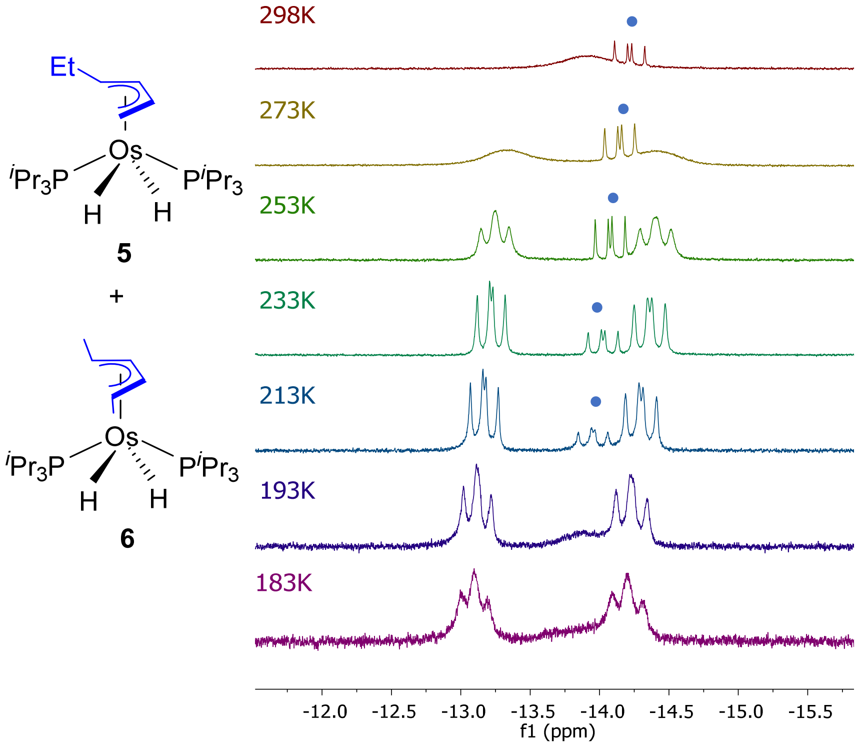

Figure S20. High-field region of the ^{1}H NMR (300.13 MHz, C_7D_8) spectrum of complexes **5** and **6** (●) between 298 and 183 K.

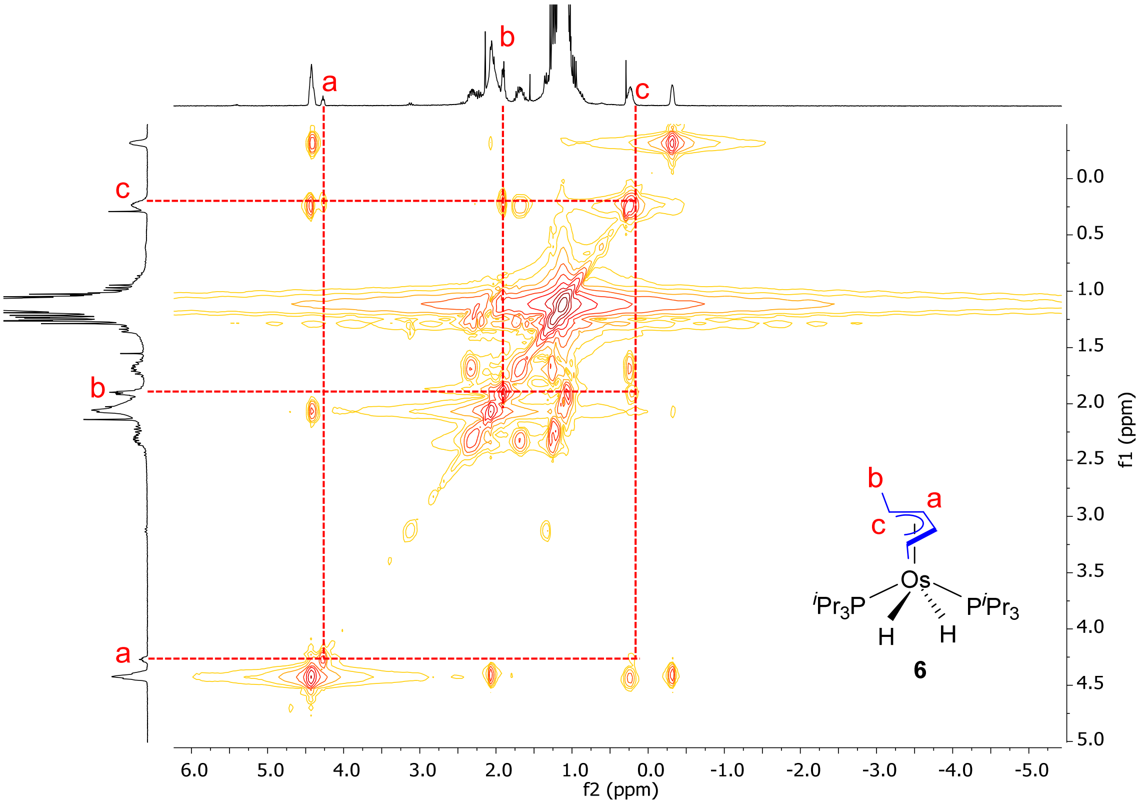

Figure S21. COSY {^{1}H} NMR (300.13 MHz, C_7D_8, 298 K) spectrum of complexes **5** and **6.** Red lines show the correlations of the hydrogen atoms of the butenediyl ligand of the minor isomer **6**.

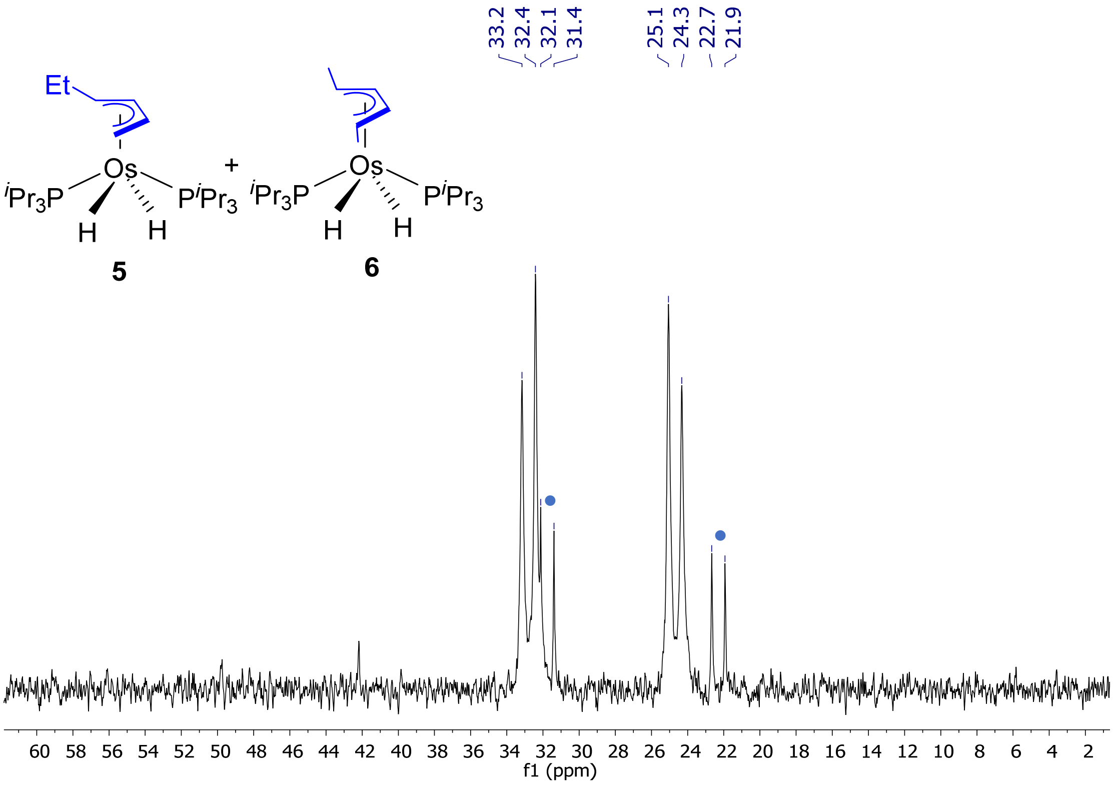

Figure S22. ^{31}P {^{1}H} NMR (121.50 MHz, C_7D_8, 253 K) spectrum of complexes **5** and **6** (●).

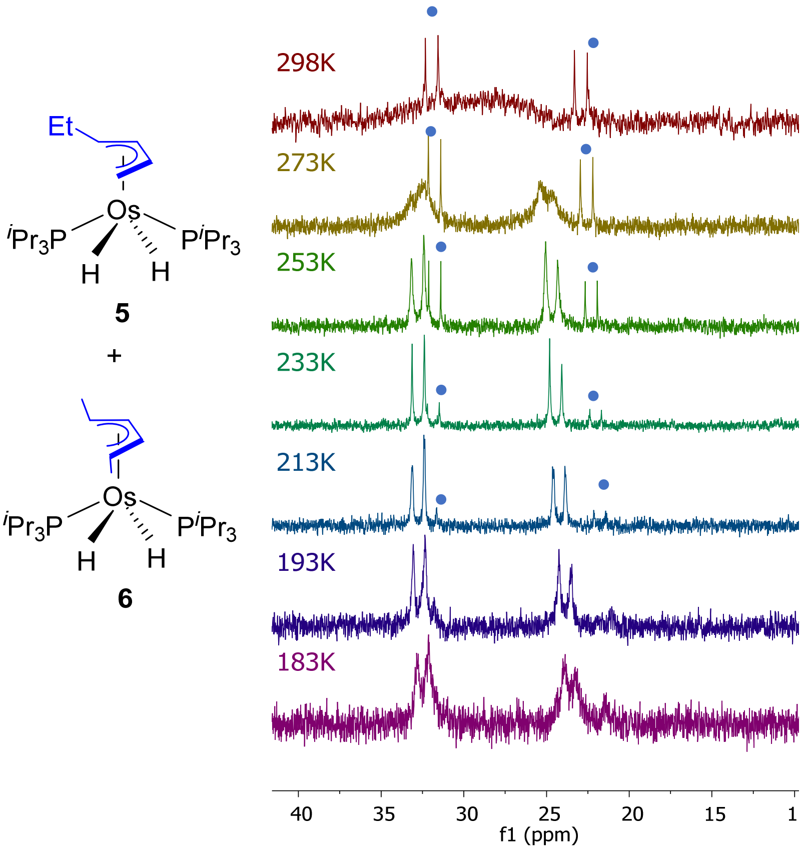

Figure S23. $^{31}P\{^{1}H\}$ NMR (400.13 MHz, C_7D_8) spectrum of complexes **5** and **6** (●) between 298 and 183 K.

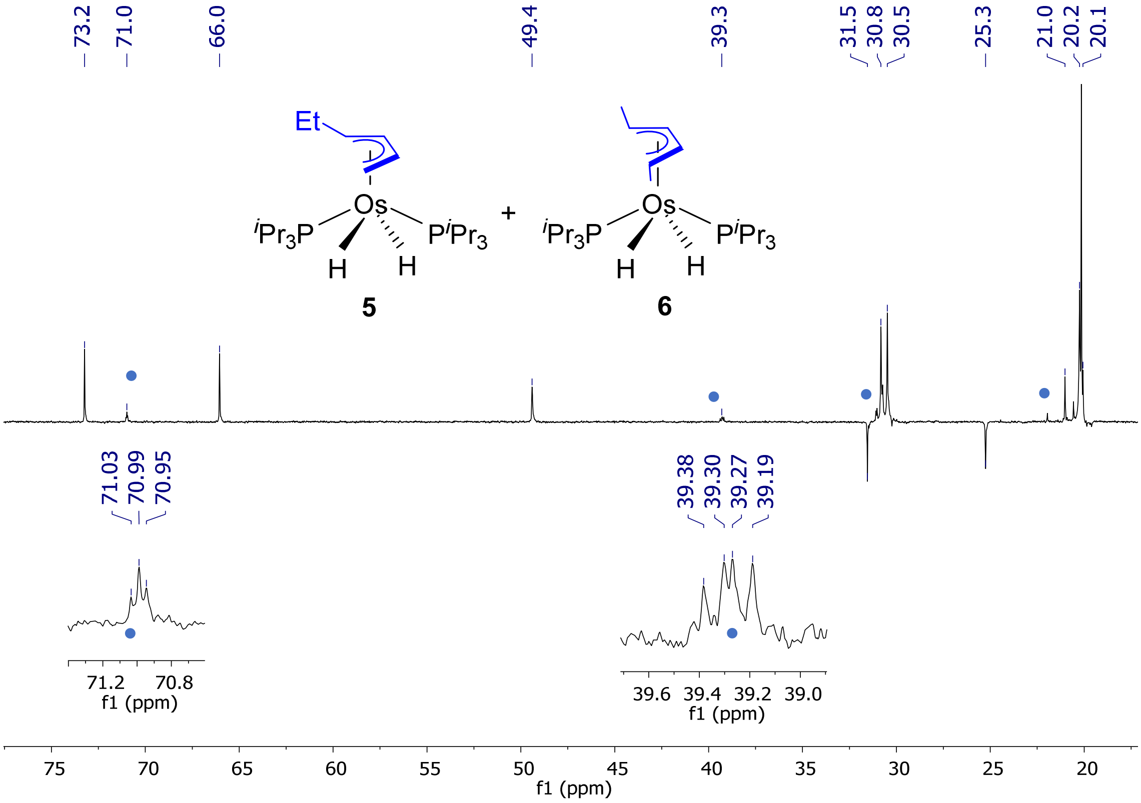

Figure S24. $^{13}C\{^{1}H\}$ APT NMR (75.48 MHz, C_6D_6, 298 K) spectrum of complexes **5** and **6** (●).

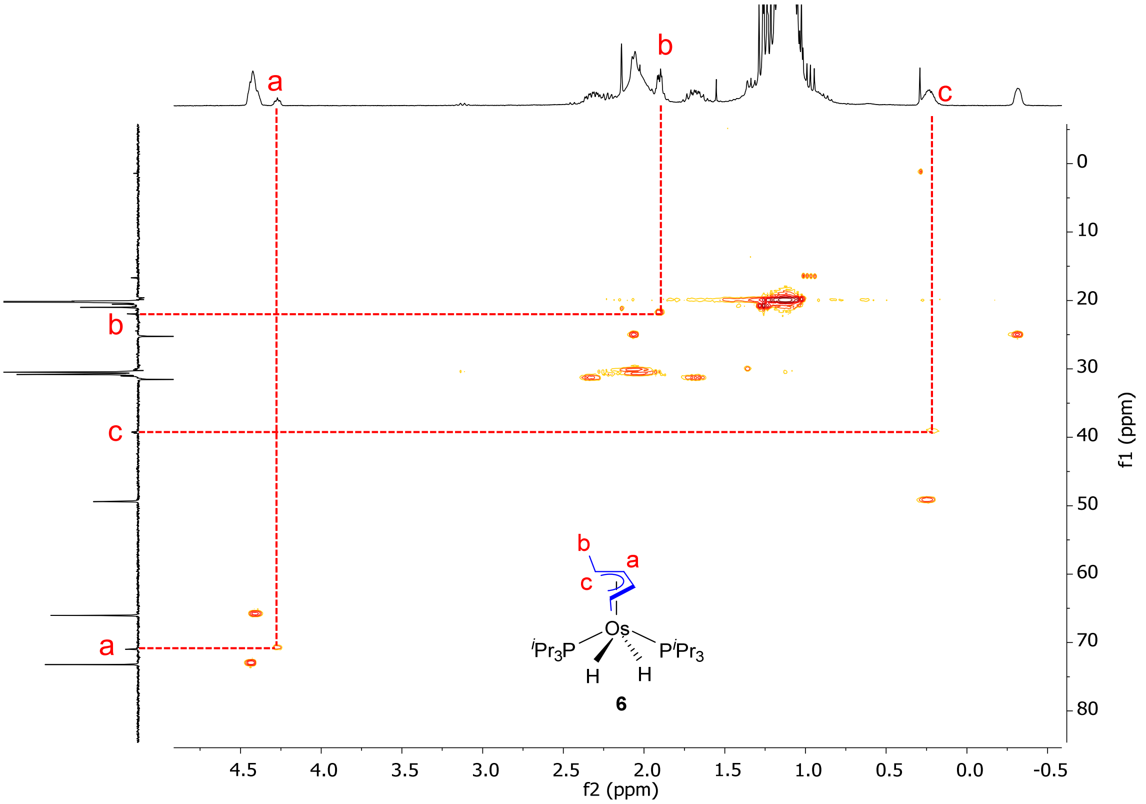

Figure S25. HSQC {^{1}H, ^{13}C} NMR (300.13, 75.47 MHz, C_7D_8, 298 K) spectrum of complexes **5** and **6.** Red lines show the correlations between the hydrogen and carbon atoms of the butenediyl ligand of the minor isomer **6**.

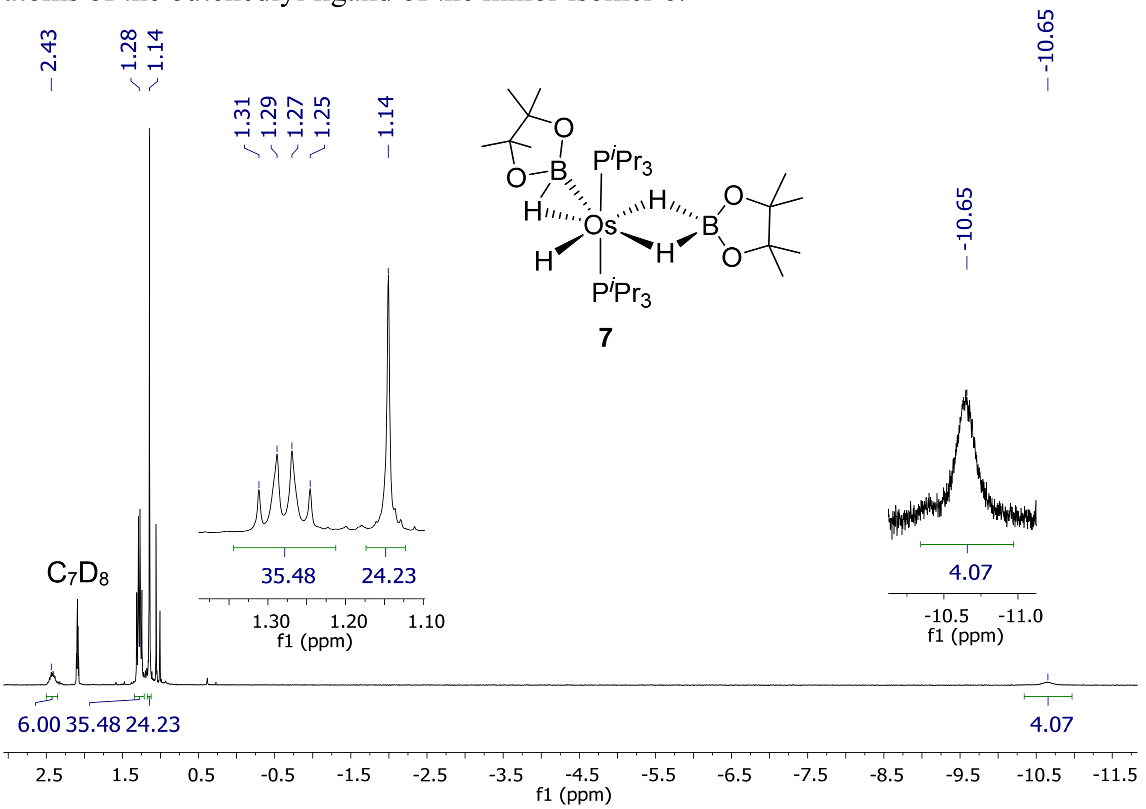

Figure S26. ^{1}H NMR (300.13 MHz, C_7D_8, 298 K) spectrum of complex **7**.

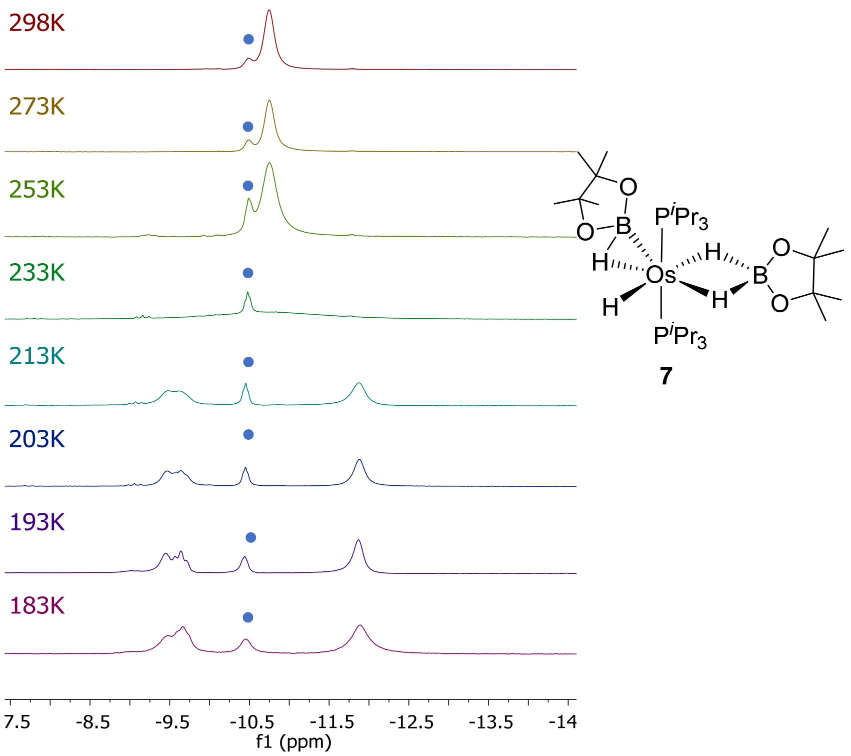

Figure S27. High-field region of the ^{1}H NMR (300.13 MHz, C_7D_8) spectrum of complex **7** between 298 and 183 K. Complex **8** (•).

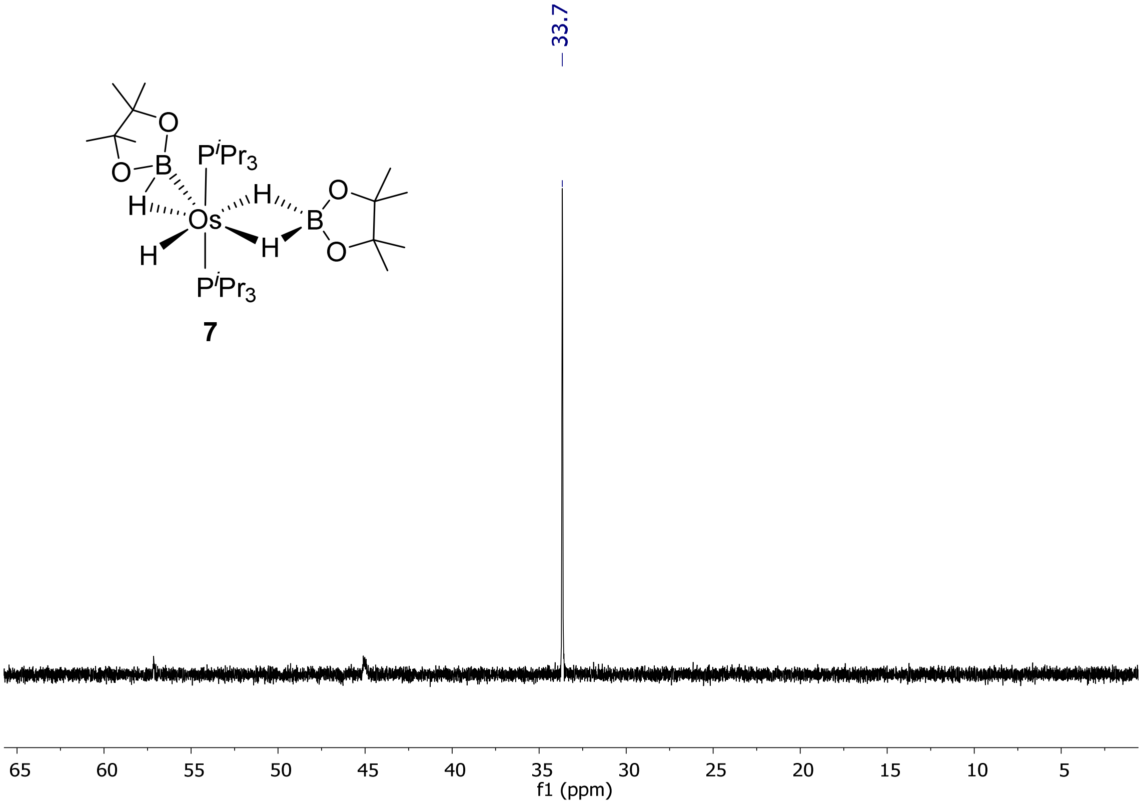

Figure S28. ^{31}P {^{1}H} NMR (121.50 MHz, C_7D_8, 298 K) spectrum of complex **7**.

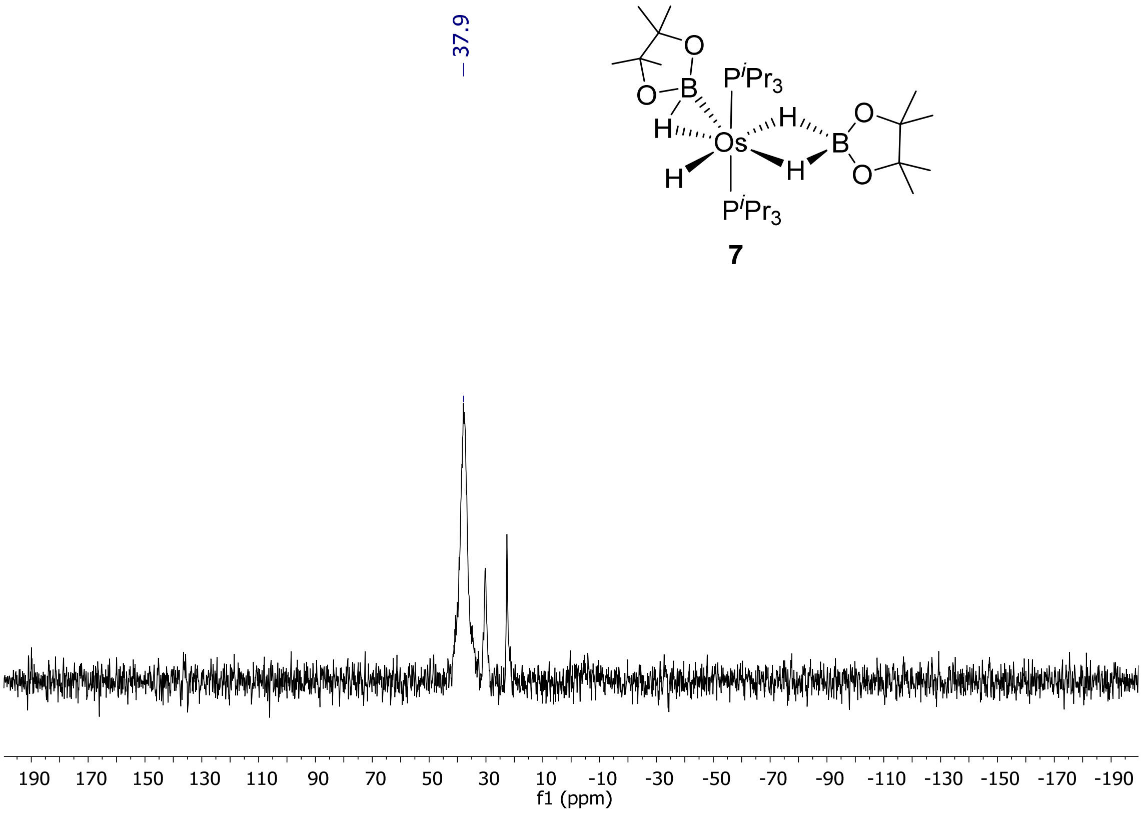

Figure S29. ^{11}B NMR (96.29 MHz, C_7D_8, 298 K) spectrum of complex **7**.

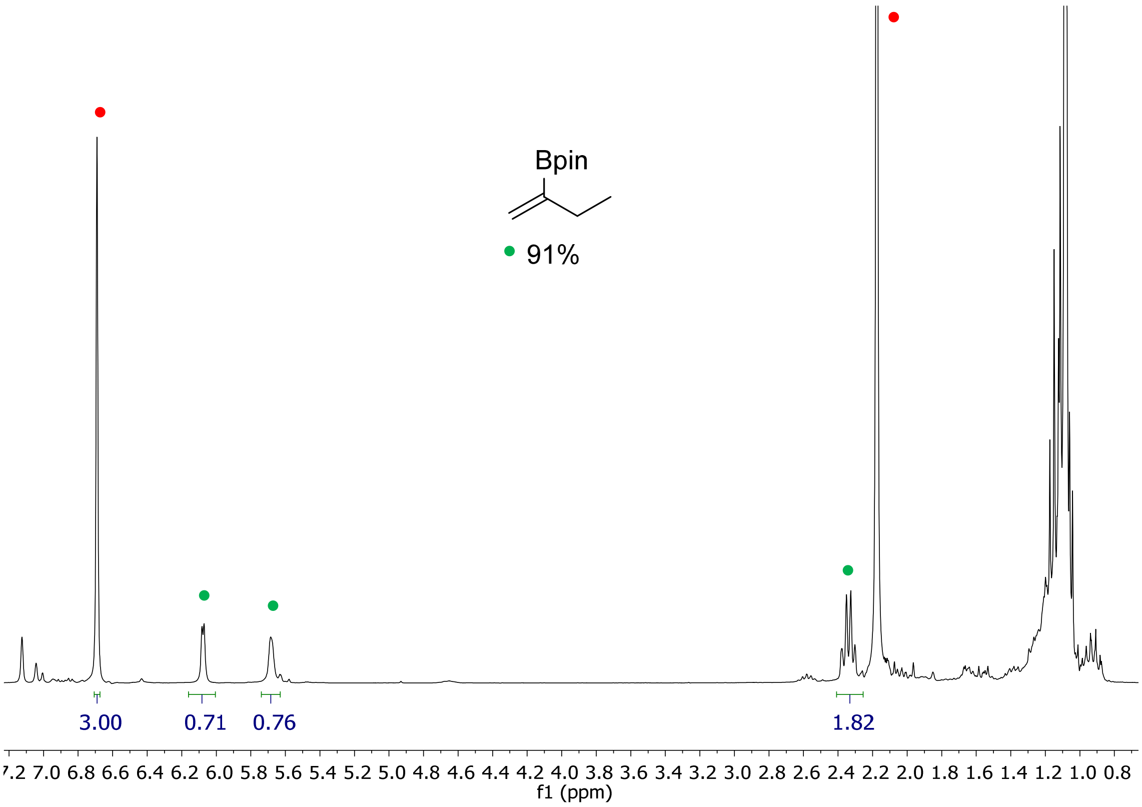

Figure S30. ^{1}H NMR (300.13 MHz, C_7D_8, 298 K) spectrum of the reaction crude of the hydroboration of 2-butyne to 2-pinacolboryl-1-butene (●) after 2 h. Mesitylene (●).

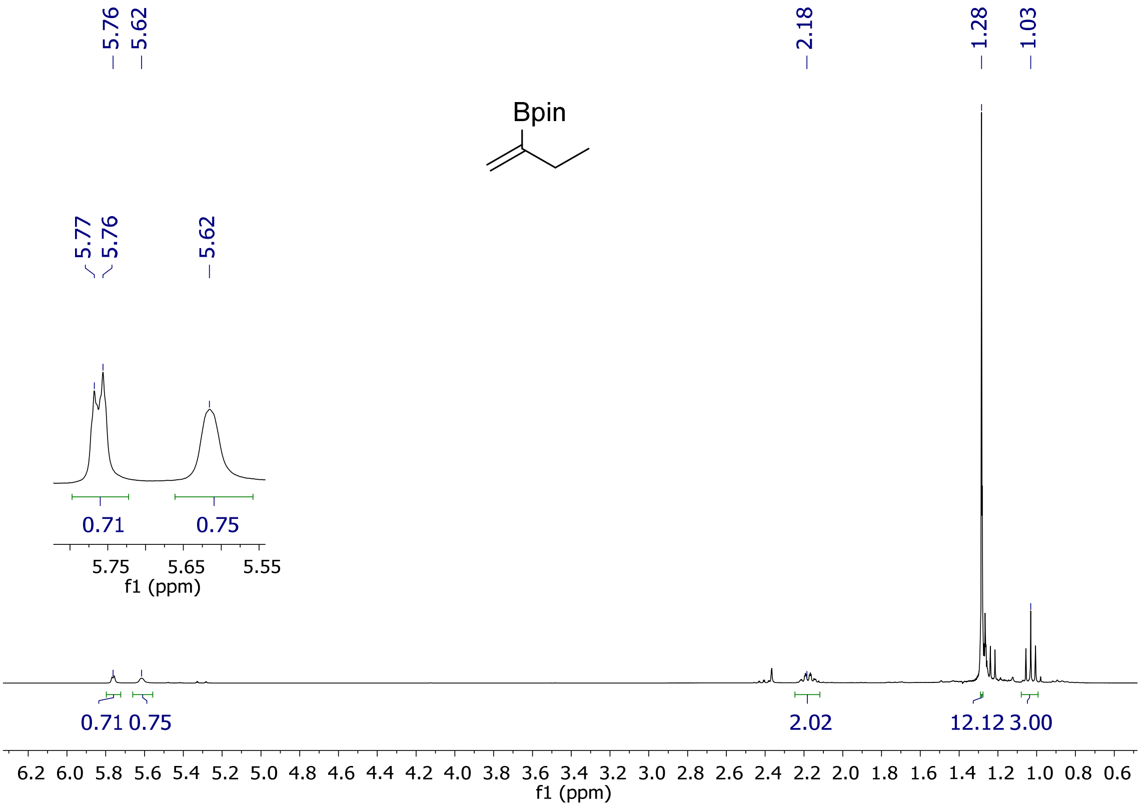

Figure S31. ^{1}H NMR (300.13 MHz, $CDCl_3$, 298 K) spectrum of 2-pinacolboryl-1-butene.

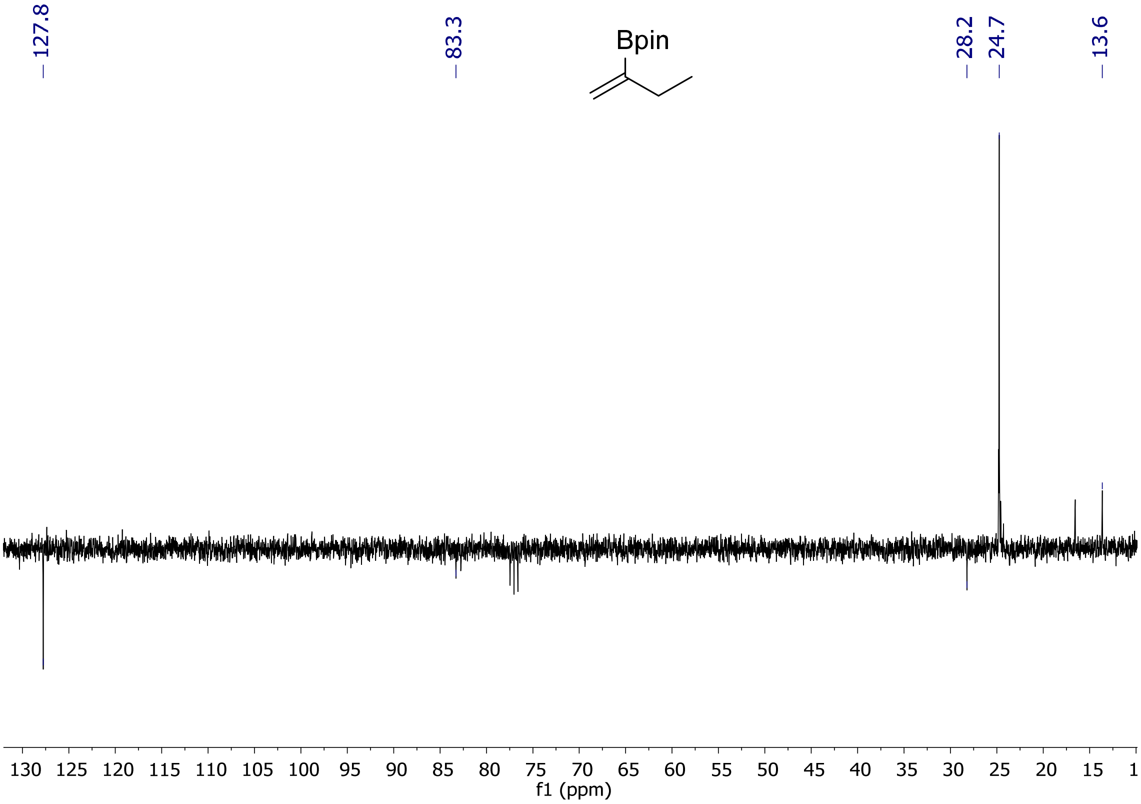

Figure S32. $^{13}C\{^{1}H\}$ APT NMR (75.48 MHz, $CDCl_3$, 298 K) spectrum of 2-pinacolboryl-1-butene.

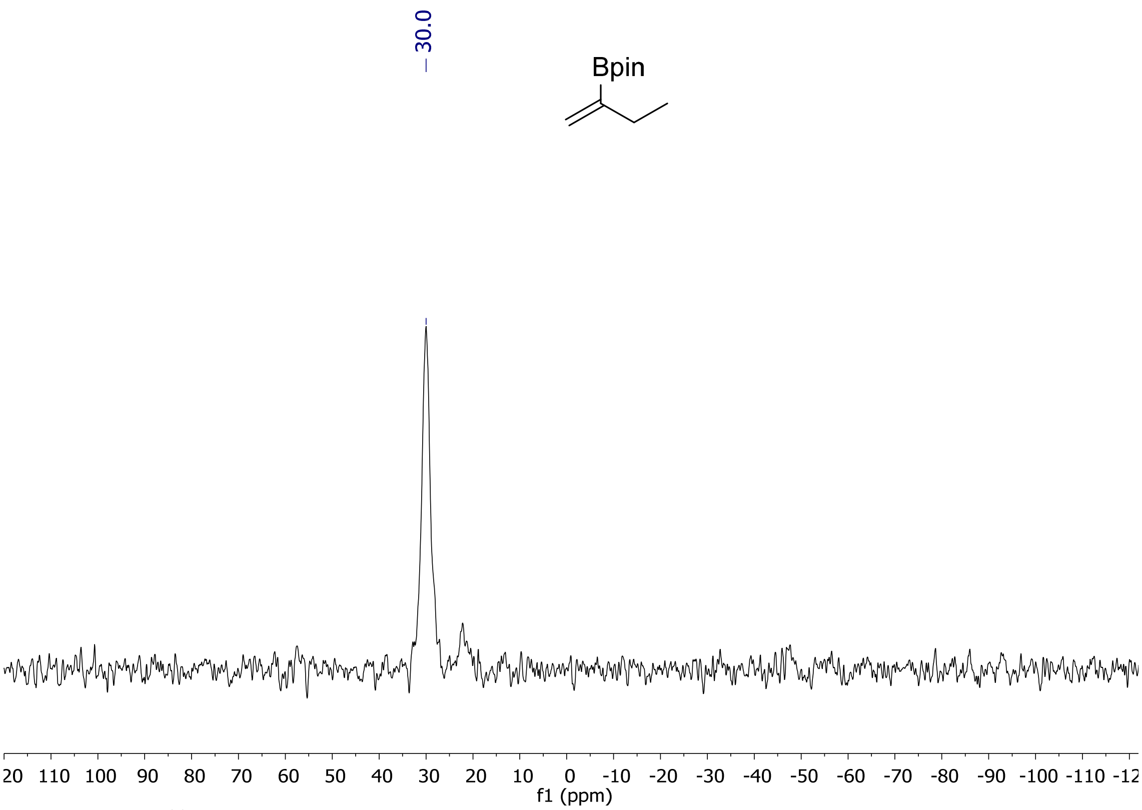

Figure S33. ^{11}B NMR (96.29 MHz, $CDCl_3$, 298 K) spectrum of 2-pinacolboryl-1-butene.

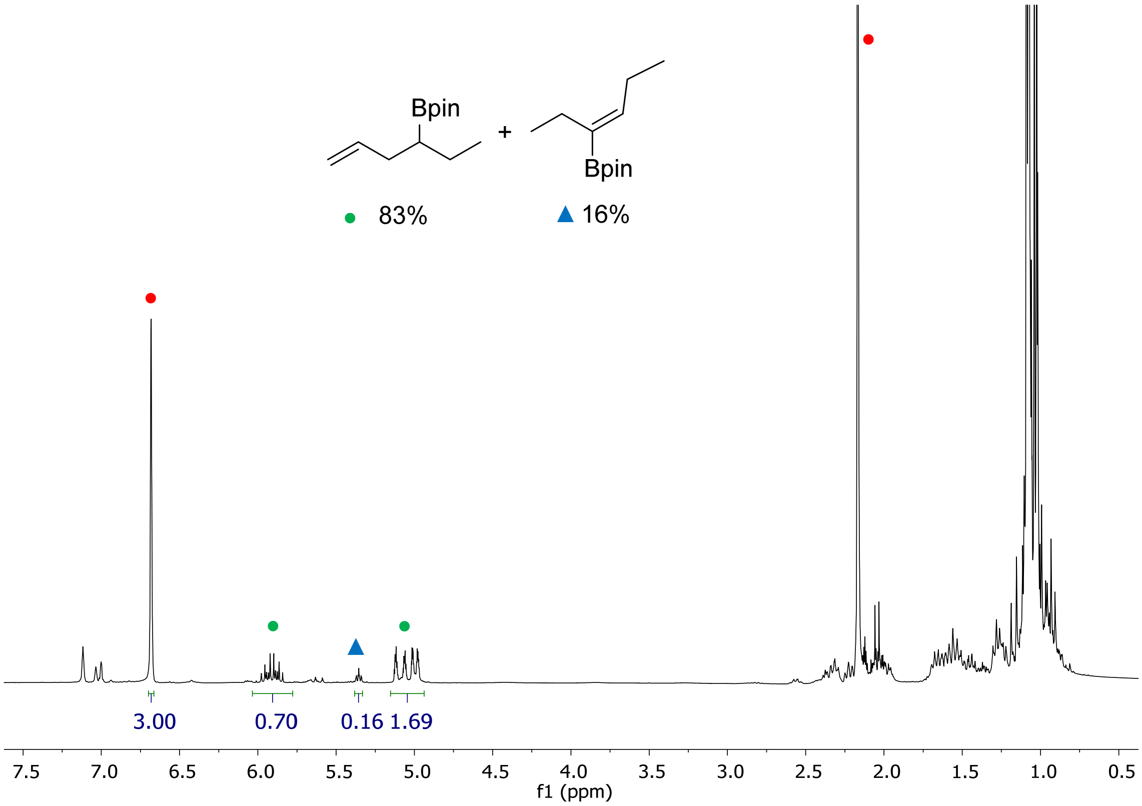

Figure S34. ^{1}H NMR (300.13 MHz, C_7D_8, 298 K) spectrum of the reaction crude of the hydroboration of 3-hexyne to 4-pinacolboryl-1hexene (●) after 3 h. Mesitylene (●). 3-Pinacolboryl-3-hexene (▲).

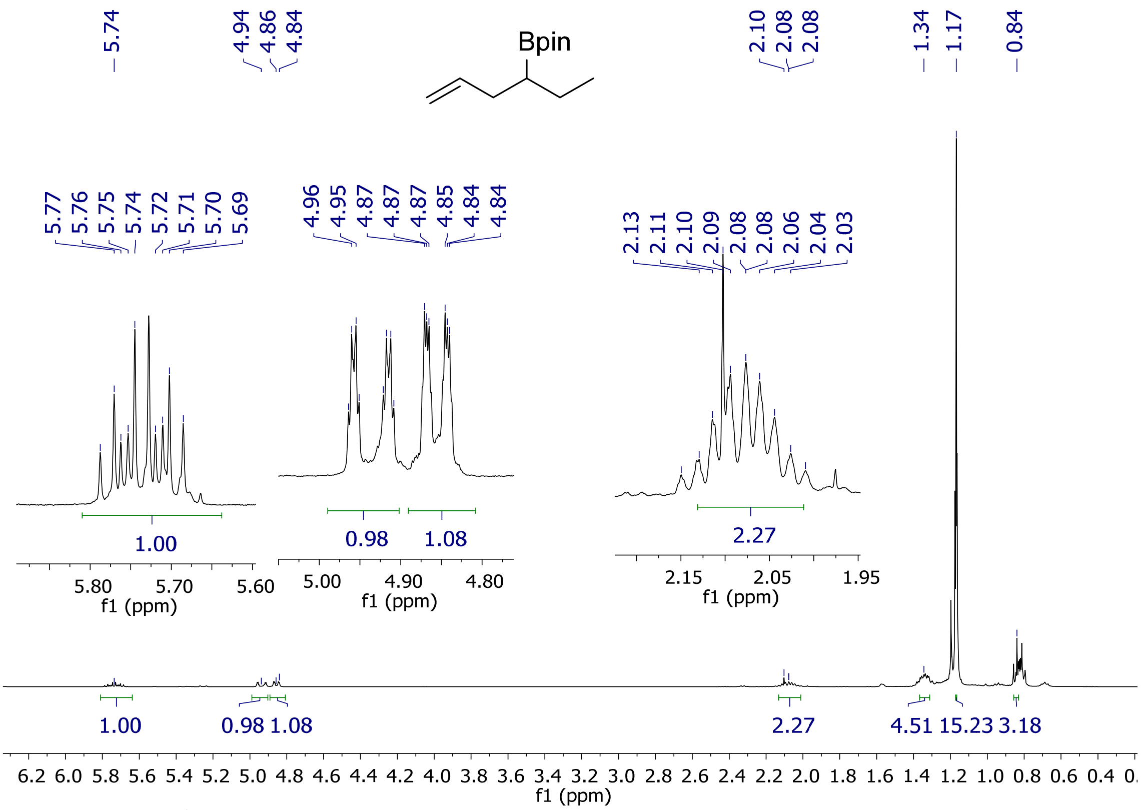

Figure S35. ^{1}H NMR (300.13 MHz, $CDCl_3$, 298 K) spectrum of 4-pinacolboryl-1-hexene.

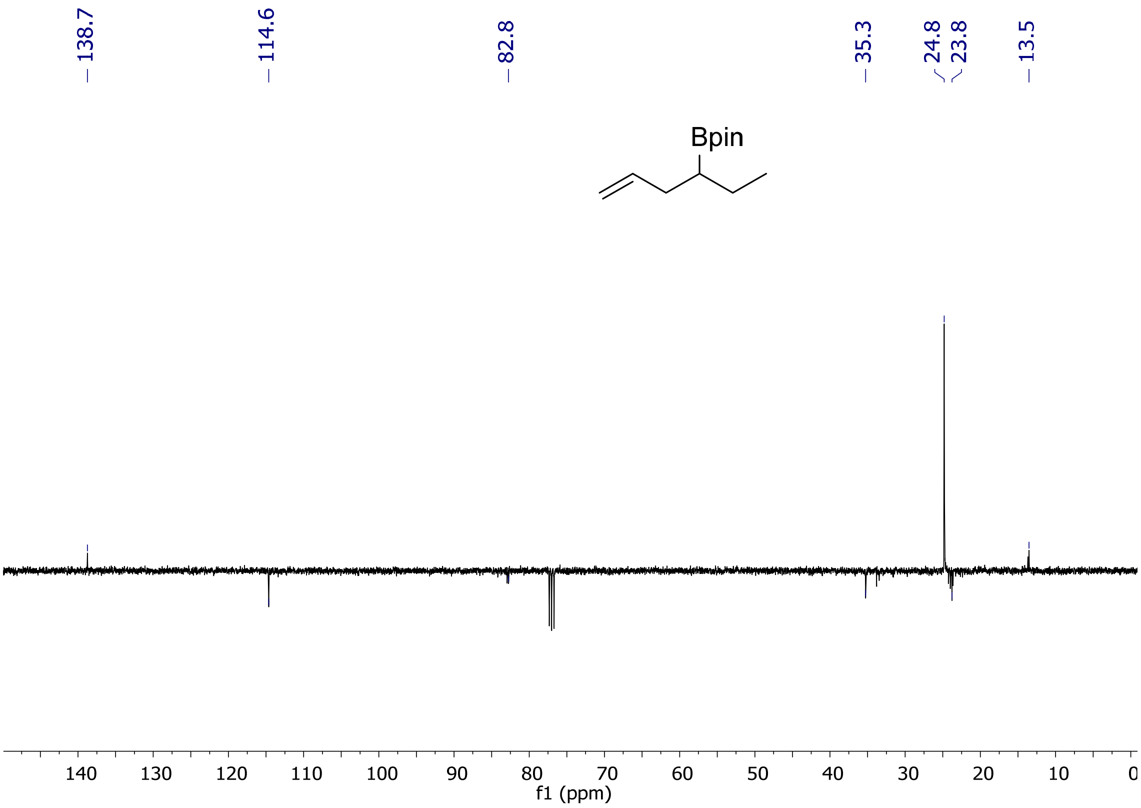

Figure S36. $^{13}C\{^{1}H\}$ APT NMR (75.48 MHz, $CDCl_3$, 298 K) spectrum of 4-pinacolboryl-1-hexene.

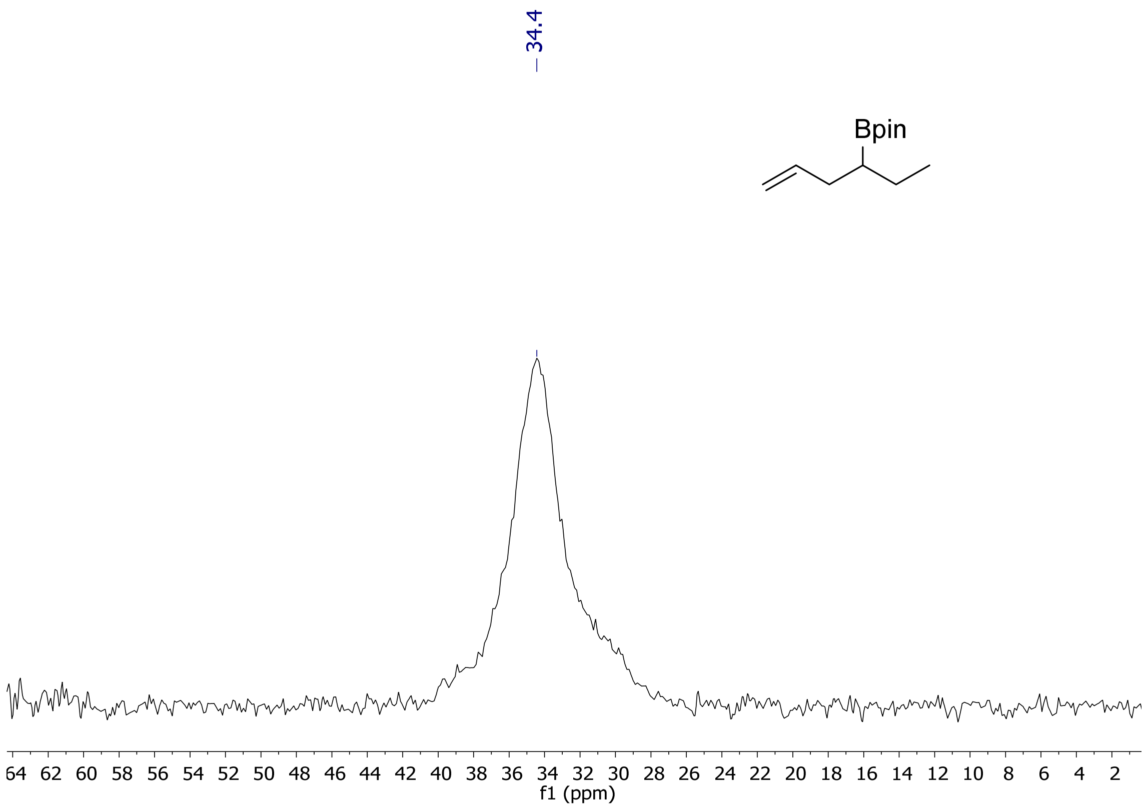

Figure S37. ^{11}B NMR (96.29 MHz, $CDCl_3$, 298 K) spectrum of 4-pinacolboryl-1-hexene.

References

(1) Aracama, M.; Esteruelas, M. A.; Lahoz, F. J.; López, J. A.; Meyer, U.; Oro, L. A.; Werner, H. Synthesis, Reactivity, Molecular Structure, and Catalytic Activity of the Novel Dichlorodihydridoosmium(IV) Complexes $OsH_2Cl_2(PR_3)_2$ (PR_3 = P-i-Pr_3, PMe-t-Bu_2). *Inorg. Chem.* **1991**, *30*, 288−293.

(2) Cancela, L.; Esteruelas, M. A.; López, A. M.; Oliván, M.; Oñate, E.; San-Torcuato, A.; Vélez, A. Osmium- And Iridium-Promoted C-H Bond Activation of 2,2′-Bipyridines and Related Heterocycles: Kinetic and Thermodynamic Preferences. *Organometallics* **2020**, *39*, 2102–2115.

(3) Blessing, R. H. *Acta Crystallogr.* **1995**, *A51*, 33. SADABS: Area-detector absorption correction; Bruker- AXS, Madison, WI, 1996.

(4) SHELXL-2016/6. Sheldrick, G. M. *Acta Cryst.* **2008**, *A64*, 112-122.

(5) (a) Lee, C.; Yang, W.; Parr, R. G. Development of the Colle-Salvetti correlation-energy formula into a functional of the electron density. *Phys. Rev. B* **1988**, *37*, 785–789. (b) Becke, A. D. Density-functional exchange-energy approximation with correct asymptotic behavior. *J. Chem. Phys.* **1993**, *98*, 5648–5652. (c) Stephens, P. J.; Devlin, F. J.; Chabalowski, C. F.; Frisch, M. J. *Ab Initio* Calculation of Vibrational Absorption and Circular Dichroism Spectra Using Density Functional Force Fields. *J. Phys. Chem.* **1994**, *98*, 11623–11627.

(6) Grimme, S.; Antony, J.; Ehrlich, S.; Krieg, H. A consistent and accurate *ab initio* parametrization of density functional dispersion correction (DFT-D) for the 94 elements H-Pu. *J. Chem. Phys.* **2010**, *132*, 154104.

(7) Gaussian 09, Revision D.01, Frisch, M. J.; Trucks, G. W.; Schlegel H. B.; Scuseria, G. E.; Robb, M. A.; Cheeseman, J. R.; Scalmani, G.; Barone, V.; Mennucci, B.; Petersson, G. A.; Nakatsuji, H.; Caricato, M.; Li, X.; Hratchian, H. P.; Izmaylov, A. F.; Bloino, J.; Zheng, G.; Sonnenberg, J. L.; Hada, M.; Ehara, M.; Toyota, K.; Fukuda, R.; Hasegawa, J.; Ishida, M.; Nakajima, T.; Honda, Y.; Kitao, O.; Nakai, H.; Vreven, T.; Montgomery, J. A.; Peralta, Jr., J. E.; Ogliaro, F.; Bearpark, M.; Heyd, J. J.; Brothers, E.; Kudin, K. N.; Staroverov, V. N.; Keith, T.; Kobayashi, R.; Normand, J.; Raghavachari, K.; Rendell, A.; Burant, J. C.; Iyengar, S. S.; Tomasi, J.; Cossi, M.; Rega, N.; Millam, J. M.; Klene, M.; Knox, J. E.; Cross, J. B.; Bakken, V.; Adamo, C.; Jaramillo, J.; Gomperts, R.; Stratmann, R. E.; Yazyev, O.; Austin, A. J.; Cammi, R.; Pomelli, C.; Ochterski, J. W.; Martin, R. L.; Morokuma, K.; Zakrzewski, V. G.; Voth, G. A.; Salvador, P.; Dannenberg, J. J.; Dapprich, S.; Daniels, A. D.; Farkas, O.; Foresman, J. B.; Ortiz, J. V.; Cioslowski, J.; Fox, D. J. Gaussian, Inc., Wallingford CT, 2013.

(8) Andrea, D.; Häußermann, U. M.; Dolg, M.; Stoll, H.; Preuss, H. Energy-adjusted *ab initio* pseudopotentials for the second and third row transition elements. *Theor. Chim. Acta* **1990**, *77*, 123–141.

(9) Ehlers, A. W.; Bohme, M.; Dapprich, S.; Gobbi, A.; Hollwarth, A.; Jonas, V.; Kohler, K. F.; Stegmann, R.; Veldkamp, A.; Frenking, G. A set of f-polarization functions for pseudo-potential basis sets of the transition metals Sc-Cu, Y-Ag and La-Au. *Chem. Phys. Lett.* **1993**, *208*, 111–114.

(10) (a) Hehre, W. J.; Ditchfield, R.; Pople, J. A. Self-Consistent Molecular Orbital Methods. XII. Further Extensions of Gaussian-Type Basis Sets for Use in Molecular Orbital Studies of Organic Molecules. *J. Chem. Phys.* **1972**, *56*, 2257–2261. (b) Francl, M. M.; Pietro, W. J.; Hehre, W. J.; Binkley, J. S.; Gordon, M. S.; DeFrees, D. J.; Pople, J. A. Self-consistent molecular orbital methods. XXIII. A polarization-type basis set for second-row elements. *J. Chem. Phys.* **1982**, *77*, 3654–3665.

(11) Zhao, Y.; Schultz, N. E.; Truhlar, D. G. Design of Density Functionals by Combining the Method of Constraint Satisfaction with Parametrization for S22 Thermochemistry, Thermochemical Kinetics, and Noncovalent Interactions. *J. Chem. Theory Comput*. **2006**, *2*, 364–382.

(12) Weigend, F.; Ahlrichs, R. Balanced basis sets of split valence, triple zeta valence and quadruple zeta valence quality for H to Rn: Design and assessment of accuracy. *Phys. Chem. Chem. Phys*. **2005**, *7*, 3297–3305.

(13) Keith, T. A. AIMAll, 2010. http://tkgristmill.com.
